# Tools for a systematic appraisal of integrated community-based approaches to prevent childhood obesity

**DOI:** 10.1186/s12889-018-5042-4

**Published:** 2018-01-29

**Authors:** K. Mantziki, C. M. Renders, M. J. Westerman, J. Mayer, J. M. Borys, J. C. Seidell

**Affiliations:** 1 Department of Health Sciences, VU University of Amsterdam, De Boelelaan 1085, 1081HV Amsterdam, The Netherlands; 2 EPODE International Network, 109-111 Rue Royale, 1000 Brussels, Belgium

**Keywords:** Evaluation, Programmes, Obesity prevention, Community, Epode, Good practice appraisal tool

## Abstract

**Background:**

Evaluation and monitoring methods are often unable to identify crucial elements of success or failure of integrated community-wide approaches aiming to tackle childhood overweight and obesity, yet difficult to determine in complex programmes. Therefore, we aimed to systematically appraise strengths and weaknesses of such programmes and to assess the usefulness of the appraisal tools used.

**Methods:**

To identify strengths and weaknesses of the integrated community-based approaches two tools were used: the *Good Practice Appraisal tool for obesity prevention programmes, projects, initiatives and intervention (GPAT)*, a self-administered questionnaire developed by the WHO; and the *OPEN tool*, a structured list of questions based on the EPODE theory, to assist face-to-face interviews with the principle programme coordinators. The strengths and weaknesses of these tools were assessed with regard to practicalities, quality of acquired data and the appraisal process, criteria and scoring.

**Results:**

Several strengths and weaknesses were identified in all the assessed integrated community-based approaches, different for each of them. The *GPAT* provided information mostly on intervention elements whereas through the *OPEN tool* information on both the programme and intervention levels were acquired.

**Conclusion:**

Large variability between integrated community-wide approaches preventing childhood obesity in the European region was identified and therefore each of them has different needs. Both tools used in combination seem to facilitate comprehensive assessment of integrated community-wide approaches in a systematic manner, which is rarely conducted. Nonetheless, the tools should be improved in line to their limitations as recommended in this manuscript.

**Electronic supplementary material:**

The online version of this article (10.1186/s12889-018-5042-4) contains supplementary material, which is available to authorized users.

## Background

Overweight and obesity are nowadays characterised as a major public health problem worldwide and are therefore highly prioritised on the European public health agenda as well [1]. The causal pathways that drive the increase of obesity prevalence are complex and predominately associated with lifestyle behaviours such as low levels of physical activity, sedentary lifestyles and unhealthy dietary habits. These lifestyles are influenced by societal, cultural, economic, organizational and environmental conditions [1–5]. This implies the need for integrating multiple sectors and targeting multiple levels of influence of unhealthy dietary and physical activity habits simultaneously [1, 5–7]. Therefore, a socio-ecological approach for interventions and programs has been proposed [5, 6, 8, 9] which involves a range of factors that affect individual behaviour, reflected at the interpersonal, organisational, community and policy levels [3, 6, 8, 9].

Based on a socio-ecological approach, integrated community-based approaches (ICBAs) arise as considered to be the most promising in tackling overweight and obesity [1]. They are composed of a cluster of strategies performed in a community, consisting of changes in the political, physical, sociocultural or economic environment, designed for individual behavioural change towards a healthier lifestyle by means of involving various institutions, organizations and local stakeholders [10]. According to the World Health Organisation [11] and various researchers, such programmes can reduce obesity [8, 12, 13], although there is still limited evidence demonstrating their effectiveness [8, 14–17]. As only a few process evaluations are carried out in combination with an effect evaluation [9, 18], the results on effectiveness cannot be attributed solely to the integrated approach. Thus, it is still unclear what are the effective elements of such integrated community-based approaches. Moreover, it is necessary to conduct effect evaluation in addition to process evaluation, in order to verify whether an improvement in the implementation process does indeed lead to a better effect. However, for creating the largest impact of ICBAs it seems important to firstly optimize the implementation so.

One of the few promising integrated community-wide approaches that have provided some insights is based on the EPODE (‘Ensemble Prévenons l’Obésité Des Enfants’ or ‘Together let’s prevent childhood obesity’) methodology*,* which depends on four main pillars: (i) political commitment, (ii) supporting services for design and implementation of interventions and campaigns (or social marketing), (iii) public and private partnerships (PPPs) and (iv) scientific monitoring, evaluation and dissemination of the programme [18, 19]. The strong political commitment refers to the official involvement of political representatives, who are in key positions for influencing local or national policies, as well as influencing relevant environmental factors that affect weight-related behaviours. Social marketing is comprised of applying marketing strategies, to achieve behavioural goals that promote health. Its messages are included into strategies, targeting the children, families and their local microenvironment, aiming at the same time to mobilise local stakeholders (teachers, catering services etc). The PPPs are established as collaboration between the academic world, the public sector-agencies and governmental institutions- and the for-profit sector, ensuring mutual respect, trust for each party and common goals. The scientific evaluation of the EPODE program includes four levels: the central organisation, the local organisation, the action at settings and the effect on the child. Consequently, the evaluation includes monitoring of process, as well as outcome indicators at all levels. The term dissemination refers to the use of evidence acquired from various sources to evaluate the implementation of EPODE and to facilitate the process evaluation. Detailed information about the EPODE philosophy and pillars can be found elsewhere [18, 19].

### The OPEN project

As an innovative framework in the field of integrated community-wide approaches for the prevention of childhood obesity, the EPODE methodology has been widely adapted by integrated community-based programmes across Europe and elsewhere, adjusted to the country’s specificities and dynamics. In 2014 a European network of integrated community-based approaches targeting childhood obesity prevention-called the OPEN (Obesity Prevention through European Network) project- was initiated with financial support of the European Union. The purpose was to improve the methods of community-wide approaches by building capacity through experience sharing and training according to the EPODE methodology, besides learning from their own strengths and limitations. The many integrated community-wide approaches aiming to tackle and/or prevent childhood overweight and obesity across Europe share obstacles challenging their effectiveness. The lack of effectiveness could also be attributed to unsuitable evaluation and monitoring methods, unable to identify crucial elements of success or failure, yet difficult to determine in complex programmes [7, 20, 21]. A systematic appraisal of the programmes’ strengths and weaknesses – namely, a detailed assessment of the steps involved and the elements that affect the various process during programme planning and implementation – would potentially enhance understanding of important programme components to be improved or to be paradigmatic.

Therefore the aims of this study were:

1. To appraise the methods of the integrated community-based approaches in a systematic way.
2. To describe the strengths and weaknesses of the appraisal tools used to achieve the first aim.

## Methods

For the systematic appraisal, two structured tools were used to identify strengths and weaknesses of the ICBAs (referred as “programme-level”). The tools are the “Good Practice Appraisal tool for obesity prevention programmes, projects, initiatives and interventions” (GPAT; Additional file 1) [22], a self-administered questionnaire of the World Health Organization and the OPEN tool (Additional file 2), a structured list of questions based on the EPODE theory, aimed to assist face-to-face interviews with the principle programme coordinators and project managers. For the second aim, the strengths and weaknesses of these tools were assessed, based on the experience of the research team in using them to appraise integrated community-based approaches, with regard to:

i. the practicalities (time, cost and burden of data collection method)
ii. the quality of acquired data (complete, clear)
iii. the appraisal process, criteria and scoring.

### Recruitment of integrated community-based approaches

We selected integrated community-based approaches programmes, initiatives and public organizations (the terms “programmes” and “integrated community-based approaches” are used alternately further in this article), which implement integrated community-based interventions to prevent childhood obesity. Inclusion criteria for the current study were that they are based in the European Union and that they were on-going programmes at the time of data collection. There was no intention to include all the existing on-going programmes of the European Union.

Two different networks of integrated community-based approaches (EPODE International Network and IDEFICS - Identification and prevention of Dietary- and lifestyle-induced health EFfects In Children and infantS) were approached. Eight programmes that were members of the EPODE International Network and three from the IDEFICS network which fulfilled the inclusion criteria were approached and accepted to participate to the OPEN project. One of the IDEFICS sites (Delmenhorst) proved not to be on-going at the time of data collection (June–September 2014) and it was therefore excluded from this analysis. Two other appropriate programmes took the initiative to participate. Thus twelve programmes were finally included into this study.

We aimed to collect information by interviewing principal coordinators and/or project managers (at the national and/or local level). The programmes varied in type. Some were programmes that used a more integrated approach involving various stakeholders, networks and settings and running for longer term; whereas others were strategies or even initiatives implementing more simple interventions or campaigns. As illustrated in Table 1, eight out of the twelve programmes were organized at the national level (i.e. in some, but not necessarily all, cities of the country), including central and local (city level) coordination with one exemption. Five were EPODE-like programmes. Another three were organized at the regional level and one of them included a central coordination team as well as a local team. One programme was organized at the local level. The programmes range from 1 to 62 communities and from school to whole-community approaches, resulting in a range of 7.000 to 300.000 children and families to be targeted/reached.

**Table 1:** Descriptive characteristics of the OPEN programmes/initiatives

Programme, *country*	Programme range (region/city)	Year of initiation	Final target group (s)	Communities/schools reached	People reached/targeted
Child health programme, *Cyprus*^a^	Regional (Nicosia)	1995	Children and their families	8 communities	4.500 children and families (incl. 1000 of the control area)
Salud Madrid, *Spain*	Regional (Madrid)^e^	N/A	Children and adolescents 0–17 years old	179 communities	1.185.156 children and adolescents
EPODE Flandre Lys, *France*^b^	Regional (Community of Municipalities French Flanders - Lys)	2004	3 months to 11 years old	8 communities	34.000 people (7000 children)
JOGG (Youngsters at a Healthy Weight), *The Netherlands*^b^	National	2010	Families with children 0–19 years old	62 communities	1.000.000 (300.000 children)
Keep fit, *Poland*	National –school based	2006	Adolescents from 13 to 15 years old	60% of the secondary schools in the country	700.000 adolescents/year
HELP (Healthy Eating Lifestyle Plan) initiative*, Malta*^c^	National	2007	Children 5–16 and families	The whole country	Whole population and at-risk population
MUNSI, *Portugal*	National	2007 (school-based pilot)	Children and teachers	1 community	1600 children
PAIDEIATROFI, *Greece*^b^	National	2008	Families with children 6–12 years old	6 communities	N/A
Good Health Partille, *Sweden*^a^	Local (Partille)	New	Children from 2 to 10 years old	1 community	7.000 children
SETS movement, *Romania*^b^	National	2011	Children from 6 to 12 years old	3 communities	190.000 children and families
Sporttube, *Slovakia*	National^d^	2009	Children from 6 to 19 years old	200 schools	53.000 children
VIASANO, *Belgium*^b^	National	2007	children 3–12 and their families	18 communities	700.000 inhabitants

^a^: Programmes included in the IDEFICS network and continues carrying out prevention activities independently

^b^: Programmes using the EPODE methodology for their realisation

^c^: HELP is a multi-level initiative running under the Health Promotion and Disease Directorate, Ministry of Energy and Health, Malta

^d^: The campaigns of the programme are implemented in different regions of Slovakia; no local coordination team involved

^e^: Although the programme is regional, it is coordinated by a central team and the actual implementation is conducted by local teams. It is considered as a two-level programme (central and local coordination)

### Development and content of the tools

A. *Good Practice Appraisal Tool*

The tool was developed under a work package of the WHO/EC DG SANCO project “Monitoring progress on improving nutrition and physical activity and preventing obesity in the EU” (2008–2010). It is an open-ended questionnaire for the systematic assessment of the quality of programmes in order to identify good practices, which could be paradigmatic for future interventions targeting obesity prevention. The GPAT was developed on the basis of outcomes from a literature review regarding evaluation criteria and assessment tools that define an intervention as effective. For pilot testing, seven programmes completed the questionnaire and provided feedback and several experts pilot tested the appraisal form by assessing independently one of the programmes, while they provided additional feedback on the tool [22].

The questionnaire is comprised of 43 questions which cover three domains:

1. main intervention characteristics
2. monitoring and evaluation of the interventions
3. implementation of the interventions.

An appraisal form is also included to calculate the score achieved for each of the items and domains assessed. Detailed information about the aim and development of the tool and the tool itself can be found elsewhere [22].

B. *OPEN tool*

For the interviews the OPEN tool was developed, a structured list of questions related to the EPODE pillars, flexible to additional information. The aims were:

1. To get insight into the way the programme was realised.
2. To identify barriers in implementation of the programmes.

The OPEN tool was developed by an expert group (i.e. the authors) – comprised by health professionals/researchers in obesity prevention and management (JCS and CR), an expert in qualitative studies (MW), professionals in development and implementation of integrated community- based interventions from the EPODE International Network (JMB and JM) and a researcher in the area of community-based interventions (KM). An expert in the evaluation of integrated community-based programmes was consulted as well. The general consensus was to use as a basis two semi-structured interview guides that have been previously used to describe the methodology of EPODE-like programmes; the “EPODE Interview Guide” developed by Van Koperen et al. [18] and the “Preliminary interview guide for the transfer of the EPODE Methodology”, developed by the EPODE International Network. After thorough assessment of and discussion about the topics of the two interview guides, the expert group developed the OPEN tool (Additional file 2).

The OPEN tool is composed of 56 questions (excluding sub-questions) exploring the four pillars of EPODE:

- The involvement and commitment of political structures and political physical persons in the programme.
- The type of public and private partnerships, if any, and their involvement in the programme.
- The methods used to design and implement interventions- including the tools, means and expertise to reach the target groups.
- The involvement of scientific expertise and methods to monitor and evaluate the programme.

Overall, the questions assess programme components either at the national and/or local level (Additional file 3). Moreover, questions regarding the interventions (*n* = 10) are included, reflecting the methods used by the programme team.

### Data collection

The principal programme coordinators and/or project managers were the main respondents to both the GPAT questionnaire and the in-person interviews. Their profession was either in disciplines of health or communication and marketing. Similarly, the profession of the other interviewees was either in health (e.g. public health specialist, clinical psychologist, paediatrician, nutritionist) or in marketing and communication.

A. *Good Practice Appraisal Tool*

The GPAT was disseminated to the principal programme coordinators through e-mail. The data collectors (i.e. the coordinators of the dissemination; KM and JM) indicated that when questions refer to interventions they should select only one in case they had multiple interventions. The completed questionnaires were reviewed on completeness and clarity. In order to ensure high quality data the data collectors discussed potential queries/misinterpretations of the questions from the GPAT face-to-face with the respondents, before the interviews through the OPEN tool were carried out. Additional information was asked (including programme documentation) and provided when necessary. Finally, verbal feedback was given from the respondents regarding the questionnaire.

B. *OPEN tool*

Face-to-face interviews with the principal programme coordinators and/or programme managers were conducted at the national or regional level (nine programmes), the local level (one programme) or both-the national and local level (two programmes). The number of the interviewees per interview ranged from 1 to 4. The same protocol was used for all programmes: the interviewers (JM and KM) visited the principal programme coordinators in their office, carried out the interviews in English language and audio-recorded them. In one case the interviewee did not speak English, thus a colleague translated the information. In addition, all questions were asked following the OPEN tool in most of the cases, whereas otherwise, the interviewers assured that all the topics had been discussed by the end of the interview. In the cases of missing or unclear information, short-term, supplementary, face-to-face interviews were conducted (*n* = 11) and additional information was asked via e-mail (*n* = 4 out of the 11 supplementary interviews).

### Data analysis

#### Appraisal of programmes’ methods by the GPAT and the OPEN tool

A. *Good Practice Appraisal Tool*

The GPAT questionnaires were appraised through the provided appraisal form (Additional file 1), which scores the items of each of the three domains of the questionnaire using a binomial scale 0 (not included element) or 1 (included element). Given that it was often difficult to decide between extreme scores, we included an intermediate scale equal to 0.5 (partly included element). After calculating the score of each section, this was divided by the maximum section score, resulting in a score of 1 or less. The score refers to “good practice” if 0.8 or higher, to “acceptable practice” when it is 0.6–0.8, to “marginal practice” when it ranges between 0.4–0.6 and to “weak practice” when it is lower than 0.4. Finally, the average score of all three sections was calculated to appraise the programme as a whole. In line with the instructions of the Good Practice Appraisal Tool [22], the data were appraised by two independent researchers. Firstly, KM made the initial appraisal. Secondly, equivocal information was thoroughly discussed with CR in order to agree on the final score of each item.

B. *OPEN tool*

The interviews were transcribed by one researcher (KM). Due to the lengthy interviews and limited time, the expert group decided to transcribe only the answers to all questions of the structured question list instead of conducting verbatim transcription. This task was carefully undertaken in order to ensure transcription of all core information.

In order to appraise the realisation of each of the programmes, the pillars of EPODE – with addition of more detailed questions formulated by the evaluators – were used as implicit benchmarks. Specifically, criteria for each of the four EPODE pillars were developed along with their scoring scales. The criteria were based on the logic model of EPODE [18] and the experience of the expert group on the critical elements of the EPODE pillars. During the appraisal process, the rating scales were adapted and criteria were added, depending on the information gathered by the programmes. This resulted in the OPEN tool analysis framework, composed by 101 items (Additional file 3). Thereafter, the information of each of the programmes was organised based on this framework, resulting in an overview of the programmes realisation (information matrix). The appraisal criteria of the analysis framework were assigned to a scoring scale from 0 to 2. A score of (0) stands for none existing element or poor quality. A score of 1 was given for existing element of moderate quality or a partly existing element and a score of 2 was given to existing elements of good quality. The reference criteria for the quality of the elements for each of the pillars, are derived by existing literature on the EPODE framework [18, 19, 23]. Three researchers (KM, CR and JM) reviewed and scored the information of each programme independently. Disagreements in the scoring of both tools were resolved by consensus of the expert group. Then a total score was calculated for each of the EPODE pillars. In many cases there were questions that did not apply to some of the programmes (labelled as “not applicable”), which were scored as 0.

During the appraisal of both sets of information, the evaluators encountered difficulties in scoring, due to essential differences in the integrated approach used by each of the programmes. Thus, interpretation of scores is dependent on the different contexts. An example is the scoring of the item about evaluation of the actions in the setting (F4biii; Additional file 2); for a local programme the score depended on whether the majority of the actions have been evaluated, but for a national programme, if the majority of the communities evaluated their actions was considered.

#### Assessment of strengths and weaknesses of the appraisal tools

In order to clarify the strengths and weaknesses of the two tools to assess integrated community-based approaches, the expert group discussed thoroughly the experience of the data collectors and evaluators in using the tools. Specifically, practical aspects of the data collection were discussed, namely the burden of the data collection method, the time and the costs needed. In addition, considering the importance of acquiring high quality data, the information collected via both tools were compared in terms of being complete and clear. Moreover, the time needed for and ease of the appraisal process were discussed, along with the appraisal criteria and scoring.

## Results

### Appraisal of the programme’s methods

A. *Good Practice Appraisal Tool*

The assessment of the programmes through the GPAT showed that their practices, covering all three domains assessed, was characterized as acceptable for the 27% (3/11 programmes) of them, as “marginal” for 54.5% (6/11 programmes) and as “weak” for 18% (2/11 programme) (Table 2). The majority of the programmes (*n* = 10) had scores below 0.60 in elements of “monitoring and evaluation” (Table 2). In the “implementation” domain most scores were between 0.18 and 0.59 (Table 2), whereas the “main intervention characteristics” domain was of moderate quality in many of the programmes (*n* = 7; Table 2). Frequent weaknesses were:

- non-inclusion of the target group in setting the objectives of the intervention, no needs assessment carried out and not addressing environmental change (main intervention characteristics)
- no measurement of process indicators (monitoring and evaluation)
- non-achievement of the majority of the objectives.

B. *OPEN tool*

**Table 2:** Scores of the programmes on the each of the GPAT’s domains

Section	Main intervention characteristics^a^	Monitoring and evaluation^b^	Implementation^c^	Total^d^
Programme, *Country*				
Child health Programme, *Cyprus*	0.66	0.31	0.41	0.45
Salud Madrid, *Spain*	0.76	0.54	0.59	0.63
EPODE (Together let’s prevent childhood obesity!) Falndre Lys, *France*	0.41	0.31	0.45	0.41
JOGG (Youngsters at a Healthy Weight), *The Netherlands*	0.95	0.61	0.41	0.66
Keep fit, *Poland*	–	–	–	–
HELP initiative, *Malta*	0.75	0	0.45	0.40
MUNSI, *Portugal*	0.81	0.50	0.54	0.61
PAIDEIATROFI, *Greece*	0.71	0.38	0.45	0.51
Good Health Partille, *Sweden*	0.71	0	0.27	0.37
SETS movement, *Romania*	0.66	0.61	0.41	0.56
Sporttube, *Slovakia*	0.39	0	0.27	0.22
VIASANO, *Belgium*	0.6	0.54	0.18	0.44
Max score	1	1	1	1
# items scored	19	13	11	43

^a^: The domain assesses the following elements: targets, relevance, sustainability, target group, partners and cooperation and planning

^b^: The domain assesses the following elements: indicators and monitoring, measurements, statistical methods, result assessment, stakeholders and communication

^c^: The domain assesses the following elements: performance, partners and cooperation, communication and documentation, target group participation and achievement of intervention objectives

^d^: Characterization of the programme practice according to the score achieved: >0.8 = “Good practice”, 0.6–0.8 = “Acceptable practice”, 0.4–0.6 = “Marginal practice”, <0.4 = “Weak practice”

The appraisal based on the four EPODE pillars showed that achievement in “political commitment” ranged from 19% to 100%, from 25% to 89% in “public-private partnerships” (PPPs), from 40% to 84% in “supporting services for implementation of interventions and campaigns” (SSIC) and from 2.5% to 70% in “scientific evaluation and dissemination” (Table 3). The results denoted several potential areas of improvement in the programmes’ approach in each of the pillars, however different for each one of them. Common weaknesses included:

- lack of signed political support and advocacy (political commitment)
- limited use of a charter describing the terms and conditions regarding the PPPs (PPPs)
- lack of target group analysis and targeting environmental change (SSIC)
- lack of an evaluation framework integrating process and effect indicators and budget allocation for evaluation (scientific evaluation and dissemination)
- poor dissemination of results (scientific evaluation and dissemination)

**Table 3:** Scores of the on each of the four EPODE pillars

Pillar	Political commitment *Score (%)*	PPPs *Score (%)*	Supporting services for implementation of interventions and campaigns *Score (%)*	Scientific evaluation and dissemination *Score (%)*
Programme, *Country*				
Child health Programme, *Cyprus*	16 (61.5)	2 (25)^a^	35 (67)	27 (67.5)^a^
Salud Madrid, *Spain*	11 (42)	7 (39)	31 (60)	22 (55)
EPODE (Together let’s prevent childhood obesity!) Falndre Lys, *France*	24 (92)	12 (67)	35 (67)	16 (40)
JOGG (Youngsters at a Healthy Weight), *The Netherlands*	22 (85)	17 (94)	42 (81)^a^	26 (65)
Keep fit, *Poland*	20 (77)	14 (78)	27 (52)^a^	23 (57.5)
HELP initiative, *Malta*	21 (81)	10 (55.5)	40 (77)	26 (65)
MUNSI, *Portugal*	17 (71)^a^	5 (28)^a^	25 (48)	28 (70)
PAIDEIATROFI, *Greece*	20 (77)	17 (94)	37 (71)	22 (55)^a^
Good Health Partille, *Sweden*	26 (100)	8 (44)	33 (63)	6 (15)^a^
SETS movement, *Romania*	15 (58)	16 (89)	38 (73)	21 (52.5)
Sporttube, *Slovakia*^b^	3 (19)^a^	5 (42)^a^	21 (40)^a^	1 (2.5)^a^
VIASANO, *Belgium*	18 (69)	13 (78)	37 (71)	18 (45)
Maximum score *Score (%)*	26 (100)	18 (100)	52 (100)	40 (100)
Number of items scored	13	9	26	23

^a^Not all items were scored; there were questions that could not be answered, because they did not apply to the programme during the appraisal

^b^The initiative consists mainly of sporadic physical activity events

### Strengths and weakness of the good practice appraisal tool

i. *Practicalities in data collection*

From the researchers’ viewpoint, the data collection through the GPAT was relatively inexpensive and time-effective, accounting for about eight man-hours (i.e. send all the questionnaires via e-mail, review their quality of information and ask clarifications). All respondents found the questionnaire too lengthy - the time to complete it ranged from 4 h to a few days - and the formulation of some questions appeared to be unclear.

ii. *Quality of data*

Eleven out of the twelve programmes returned the completed questionnaire to the researchers. Five respondents mixed answers referring to the programme with those to the intervention level, whereas six responders focused on only one level (programme or intervention). In addition, seven respondents misinterpreted the terminology of items in the domain of “evaluation and monitoring” (21–23, 27–28; Additional file 1). Furthermore, the data collectors required the programme documentation to get insight in the context, but this was often absent or not available in English for all the programmes.

iii. *Appraisal process, criteria and scoring*

The evaluators spent 1–2 h for the data appraisal per programme and the process was difficult given the confusing information retrieved, as described above. One of the appraisal criteria did not correspond to the question asked (40). The criteria of five items (9, 13, 15, 16) were vaguely defined (Additional file 1), leading to difficulties in scoring. Therefore, the evaluators appraised them often as “partly included element” (0.5). Another observation was that the appraisal of item 7 depended on the response of item 6, which in many cases was either replied inconsistently for the (intervention/programme) level (*n* = 2) or not specified (*n* = 2) or was missing/not conducted (*n* = 1/*n* = 3). Furthermore, the appraisal criteria were not formulated or suitable for the programme level.

### Strengths and weakness of the OPEN tool

i. *Practicalities of data collection*

Twelve face-to-face interviews were conducted. The data collection included considerable costs for the transportation/accommodation of the data collectors in twelve countries. Approximately 6–8 h of transportation (with return) per visit, additionally to 1,5–4 h for conducting the interviews were spent per data collector. The interviewees spent much of their time as well for the interview. Their burden decreased given the structured topic list, which facilitated clear questions and their immediate clarification by the interviewers when needed. The transcription lasted from 5 to 10 h per interview.

ii. *Quality of data*

We obtained clear and complete information on the programme and intervention level, especially after the complementary requested information (i.e. supplementary interviews, e-mails). The questions asked and responses given during the interviews were clarified when necessary, avoiding misinterpretation by the interviewees and allowing better understanding for the interviewers.

iii. *Appraisal process, criteria and scoring*

The average time of appraisal per programme was 8 h. The appraising of the programme elements was difficult, due to the amount of information, large variability of the programmes in terms of complexity and the level of independence of the communities on their national coordination. The OPEN analysis framework was developed using criteria related to the EPODE pillars and rating scales. These criteria and rating scales were being further specified during the analysis, resulting in a framework accounting for the programmes’ variability.

## Discussion

### Appraisal of integrated community-based approaches’ methods

Several strengths and weaknesses were found in all programmes, different for each of them. It is noteworthy that the quality of the elements used for the programmes’ differed per domain/pillar assessed. For example, a programme could have a high score in elements of the implementation domain, but moderate scores in elements of the monitoring and evaluation domain. Therefore, a higher score in a domain/pillar does not imply that a programme was better as a whole than another with a lower score. However, one may compare the programmes per domain (or per element), always when taking into account their variable contexts; namely i. the level of action (national, local or both) and the actions themselves, ii. the number of settings in which EPODE approach was implemented within a community (one setting VS multiple settings targeted), iii. The number of people targeted, iv. the number of communities involved and v. the level of dependence of these communities on the central coordination to run their actions.

Although it was not possible – or even correct – to compare the results of each of the programmes between the two tools, considering that they assess different elements under their themes (i.e. domains for the GPAT and pillars for the OPEN tool), some of the identified weaknesses were common in both tools. More specifically, all the programmes scored moderately to low with regard to monitoring and evaluation, and a process evaluation was poor or not carried out. Poor evaluation or absence of process evaluation is commonly observed in integrated community-based approaches, while possible explanations include the lack of motivation, resources, time and knowledge [7, 24]. Another observation through both tools was that the target group was not thoroughly assessed prior to designing the interventions. A target group analysis, is a critical component in order to get insights into the needs, wishes, strengths and talents of the target group [25]. When these elements are taken into consideration, the chances to reach, engage and achieve behavioural change of the groups in question are increased [26–29]. In this study it was also shown that only a few programmes implemented interventions targeting environmental change, despite being community-based approaches. This indicates the importance of strengthening the partnerships with key community stakeholders involved in childhood settings. As also highlighted in the report of Lobstein and colleagues, all childhood settings are important in shaping healthy environments and improving lifestyle behaviours [30].

#### Strengths and weaknesses of the appraisal tools

The OPEN tool mainly enabled the identification of strengths and weaknesses of integrated community- based approaches. The tool detected key information on both programme and intervention levels of all study objects and thus, deeper insight was provided than with the GPAT. The GPAT proved to be suitable to identify strengths and weaknesses of more simple interventions. It is well-known that traditional evaluation criteria of interventions examine its overall effectiveness, which is not suitable for complex community-based approaches [21]. Instead, evaluation methods should be sensitive in capturing the dynamics of complex approaches, which operate through multi-dimensional causal pathways*,* and account for the different roles that various people delivering interventions have and the choices they make [31–33].

Our experience in using the appraisal tools showed that it is not a simple task to evaluate a complex approach through them. The GPAT, although it seems a “user-friendly” evaluation tool, makes use of terminology and criteria that are not very clear to practitioners. Even after explaining to the programme coordinators their queries and after specifying the kind of information expected from the different questions (where the replies were unclear/insufficient), the replies were not always completely clear. Consequently, data collection through GPAT seems feasible for professionals without much experience in the evaluation field, after learning the purpose of each of the questions. However, the appraisal of a programme is not a task that practitioners lacking relevant expertise would be able to carry out without the assistance of experts. Yet, we anticipate that these issues can be outreached once the tool is improved. Similarly, the OPEN tool requires very good knowledge of the elements and/or process of complex approaches in order to collect and especially to appraise the data. The assistance of experts in evaluation and/or long-term experience in Public Health Practise is needed and highly recommended, as practitioners face difficulties in using comprehensive evaluation tools [7]. Other comprehensive tools that have been used for the process evaluation of complex interventions seem equally difficult to be used practitioners in terms of resources and required expertise, though very useful for evaluation professionals [26, 34].

The pillar of “scientific monitoring, evaluation and dissemination” of the OPEN tool included the most objective assessment elements compared to the other pillars, questioning-among others- the (type of) monitoring of processes, as well as the evaluation of effects. Such assessment is supported by evidence indicating that, besides assessing the programme’s effectiveness, an insightful process evaluation is needed to answer questions for the conceptualisation, planning and performance of the programme [18, 21, 35].

#### Strengths and limitations of the study

This is one of the few studies that appraised community- based approaches targeting obesity prevention, including the EPODE-like programmes, which have not been assessed before in a systematic way. Our innovative methodological framework combined two methods for conducting in-depth assessment of such approaches. On the one hand, face-to face interviews, using a structured list and criteria related to the EPODE pillars, successfully provided insight into crucial elements of community- based approaches, reflecting the quality of involvement of community, political, private and scientific stakeholders and of social marketing principles. This method enhanced our understanding in how complex prevention programmes could be monitored and evaluated. On the other hand, this is the first documentation on the use of the GPAT, while its applicability in appraising integrated community-based approaches is described. Furthermore, two and three researchers were in charge of conducting the appraisal through the GPAT and the OPEN tool respectively, which decreased-but not eliminated- the subjectivity of the programme appraisal.

Nevertheless, the appraisal relied on self-reported information from the programmes. Therefore information bias is possible, as widely observed in survey research, attributed to the respondent’s comprehension, recalling ability from long-term memory, judgement of the retrieved information from his/her memory and selection to report an answer [36]. Another limitation of our study was that the qualitative information was reduced to simple scores, which reduced the ability of the evaluators to interpret the programmes, without additional context information. For instance, in the cases of scoring into the category “partly included element”, this ranged from “almost not” to “almost yes”. Moreover, considering the weaknesses of the appraisal tools used in this study, all the crucial elements of an integrated community- based approach have not been assessed. In addition, the appraisal of the GPAT was done only by the researchers of the current article, whereas researchers/experts from the WHO did not participate in this process due to bureaucratic issues. Nevertheless, the experts responsible for this tool (Joao Breda; Jo Jewels) were fully informed about the results of this research and even agreed to the limitations in relevant discussions. Finally, the included integrated community-based approaches were selected through networking and therefore they are indicative rather than representative of such approaches in the European region.

## Conclusions

There is large variability between integrated community- based approaches preventing childhood obesity in the European region-even if they follow a similar approach (i.e. EPODE-like programmes)- and therefore each of them has different needs. Yet, it is evident that monitoring and evaluation methods are still not well-developed, if carried out at all. Furthermore, knowledge of the target group is limited, while environmental change is not frequently addressed in the approaches assessed in the current study. Finally, both tools we used seem to facilitate comprehensive assessment of integrated community-based approaches in a systematic manner, which is rarely conducted. Nevertheless, the tools should be improved in line to their limitations as presented in this manuscript.

### Recommendations

Based on our conclusions we suggest as regards to the appraisal tools, firstly the creation of programme documentation, which shall be available also in English, in order to be communicated more easily among stakeholders, other programmes and experts in the field of evaluation from different countries. Secondly, improving the formulation of the GPAT’s questions will increase its applicability to the programme level. These two steps would give an overview of an integrated community- based approach. As a third step, in-person interviews through the OPEN tool shall complement unclear/missing information by the GPAT and the programme documentation, while they will enhance the assessment of the programme’s methods. Consequently, the programmes will potentially be improved, while public health practice and the involved stakeholders will be better informed.

## Additional files

Additional file 1: Good Practice Appraisal Tool. (PDF 4174 kb)

Additional file 2: OPEN tool question list. (DOCX 37 kb)

Additional file 3: Analysis grid of the OPEN tool. (DOCX 56 kb)
