# Supplementary material for: Tools for a systematic appraisal of integrated community-based approaches to prevent childhood obesity
Source: BMC Public Health. 2018 Jan 29;18:189. doi: 10.1186/s12889-018-5042-4 (PMC5789618; doi:10.1186/s12889-018-5042-4)
Supplement: Supplementary file 1 — Good Practice Appraisal Tool. (PDF 4174 kb) [file 12889_2018_5042_MOESM1_ESM.pdf]

Good Practice Appraisal Tool

for obesity prevention programmes, projects, initiatives and interventions

World Health Organization
REGIONAL OFFICE FOR Europe

WHO/EC Project on monitoring progress on improving nutrition and physical activity and preventing obesity in the European Union

EUR/10/ EUDHP1003966/ 3

ABSTRACT

The World Health Organization Regional Office for Europe and the Directorate-General for Health and Consumers of the European Commission have established a joint three-year project to monitor progress in improving nutrition and physical activity and preventing obesity in the European Union.

As part of this project, a good practice appraisal tool was developed to assess good practice elements of design, monitoring, evaluation and implementation of preventive programmes, projects, initiatives and interventions that aim to counteract obesity and improve nutrition and physical activity. This report gives a description of the good practice tool for obesity prevention programmes and describes its development and use.

Keywords

BENCHMARKING
PRACTICE GUIDELINES
OBESITY - prevention and control
PROGRAM EVALUATION - methods
EUROPE

Address requests about publications of the WHO Regional Office for Europe to:
Publications
WHO Regional Office for Europe
Scherfigsvej 8
DK-2100 Copenhagen Ø, Denmark

Alternatively, complete an online request form for documentation, health information, or for permission to quote or translate, on the Regional Office web site (http://www.euro.who.int/pubrequest).

© World Health Organization 2011
All rights reserved. The Regional Office for Europe of the World Health Organization welcomes requests for permission to reproduce or translate its publications, in part or in full.

The designations employed and the presentation of the material in this publication do not imply the expression of any opinion whatsoever on the part of the World Health Organization concerning the legal status of any country, territory, city or area or of its authorities, or concerning the delimitation of its frontiers or boundaries. Dotted lines on maps represent approximate border lines for which there may not yet be full agreement.

The mention of specific companies or of certain manufacturers' products does not imply that they are endorsed or recommended by the World Health Organization in preference to others of a similar nature that are not mentioned. Errors and omissions excepted, the names of proprietary products are distinguished by initial capital letters.

All reasonable precautions have been taken by the World Health Organization to verify the information contained in this publication. However, the published material is being distributed without warranty of any kind, either express or implied. The responsibility for the interpretation and use of the material lies with the reader. In no event shall the World Health Organization be liable for damages arising from its use. The views expressed by authors, editors, or expert groups do not necessarily represent the decisions or the stated policy of the World Health Organization.

The responsibility for the content of this report lies with the authors, and the content does not represent the views of the European Commission; nor is the Commission responsible for any use that may be made of the information contained herein.

Table of Contents

List of Abbreviations 4

Acknowledgements 4

Introduction 4

Background 4

Aim of the tool 5

Development of the tool 5

The tool components 5

Scoring of good practice 6

Assessment of programmes 6

Questionnaire to gather information
on obesity prevention programmes 7

Appraisal form – a checklist for reviewers 17

References 22

List of abbreviations

The following abbreviations are used in this report.

DALY	disability-adjusted life years
DG SANCO	Directorate-General for Health and Consumers (EC)
EC	European Commission
EPODE	Ensemble prévenons l'obésité des enfants (Together let's prevent childhood obesity)
EU	European Union
NOPA	Nutrition, Obesity and Physical Activity (database)
QALY	quality-adjusted life years
RIVM	Dutch National Institute for Public Health and the Environment
WHO	World Health Organization

Acknowledgements

This document is a deliverable of work package 3 of the three-year collaborative project between the World Health Organization (WHO) and the Directorate-General for Health and Consumers (DG SANCO) of the European Commission (EC), which began in January 2008 (2007WHO02) under the title "*Monitoring progress on improving nutrition and physical activity and preventing obesity in the European Union (EU)*". A draft of the tool was discussed at a meeting on community initiatives to improve nutrition and physical activity, convened on 21–22 February 2008 in Berlin, Germany. WHO would like to express sincere appreciation to the participants of this workshop for their valuable contributions. WHO also wishes to thank the programmes' stakeholders that took part in the pilot testing rounds of the tool. WHO is most grateful to the experts that pilot tested the appraisal component of the tool to evaluate the good practice elements of the programmes: Wanda Bemelmans, Roos Gun, Tim Lobstein, Susanne Løgstrup, Alessanra Suglia and Joop van Raaij. Special thanks are due to the members of the project's Advisory Group (Michele Cecchini, Barbara Legowski and Jean-Michel Oppert) for critically reviewing the tool and to the Dutch National Institute for Public Health and the Environment (RIVM) for its technical input throughout the development of the tool. Grateful thanks are extended to Katerina Vrtikapa, former intern at the WHO Regional Office for Europe, Copenhagen and Sonja Kahlmeier, Institute for Social and Preventive Medicine, Zurich for their technical input to the development of the tool; to Lideke Middelbeek, WHO Regional Office for Europe, Copenhagen for refinement of the tool and for writing this document; to Trudy Wijnhoven, WHO Regional Office for Europe, Copenhagen for her technical input and overall coordination; to Philippe Roux, DG SANCO, Luxembourg City for his feedback; and to Frank Theakston for the text editing.

Introduction

A three-year joint WHO/EC DG SANCO project covering the period 2008–2010, entitled "*Monitoring progress on improving nutrition and physical activity and preventing obesity in the EU*" was established to evaluate the status of country development and implementation of policies and actions in the area of nutrition, physical activity and obesity prevention. The main outcome of the project is a database on these areas (the NOPA database), which includes surveillance data, country policy documents, policy implementation tools and information on good practices. Work package of this project concerns the collection of existing public health programmes, projects, initiatives and interventions[1] designed to improve nutrition and physical activity or prevent obesity of the general population. Another important component is the development of a good practice appraisal tool to review and assess the quality of the identified programmes by independent experts. Both a summary of the programmes and an indication of good practice will be made available through the NOPA database.

This report describes the development of the appraisal tool, presents its three components and gives instructions on how the Regional Office will use it.

Background

Overweight and obesity are serious public health challenges in the WHO European Region *(1)*. Many local and national programmes aim at counteracting the increasing obesity levels by promoting healthy eating and physical activity *(2)*. Some of these programmes have shown to be more successful than others in preventing obesity and thus can serve as good examples for programme planners and decision-makers in order to facilitate their choice of interventions to adopt. To identify good practice, a tool has been developed to evaluate good practice elements of the planning, monitoring, evaluation and implementation of programmes that can target children, adolescents or adults as well as be nationally, regionally or locally initiated in community, school or workplace settings.

[1] Hereinafter, the term programmes refers to programmes, projects, initiatives and interventions.

Aim of the tool

The purpose of the tool is to systematically assess the quality of programmes. Using a set of predefined criteria, the tool aims to identify programmes that can be considered good practice and can serve as an example for future initiatives that aim to improve nutrition and physical activity or prevent obesity. The tool can be used to monitor and document the aspects of the programmes that are known to contribute to the effectiveness of an intervention and to identify points for improvement. The tool generates a good practice score for three different programme components (planning, monitoring and evaluation, and implementation) as well as for the intervention as a whole.

Development of the tool

The following methods were employed in developing the tool.

1. A literature review was carried out on evaluation criteria for determining the effectiveness of interventions, assessment tools for obesity and public health interventions and scoring systems *(3-15)*. The outcome of this review resulted in a first set of quality criteria that may be regarded as predictors of good practice and in a first draft of the tool.
2. In February 2008, the Regional Office organized a meeting on community interventions to improve nutrition and physical activity, which was hosted by the German Federal Ministry of Health *(2)*. During the meeting, a consultation round was organized to discuss different elements of community interventions and to get feedback on the first draft of the tool. The received comments were used to further refine the tool. In addition, some experts were consulted individually.
3. To identify gaps and to review feasibility, user friendliness and relevance, the tool was pilot tested through three pilot rounds between 2007 and 2009. Eleven programmes were approached to complete the questionnaire (first component of the tool), provide relevant reference material and give feedback on the questions included. Feedback was received from seven:
   - "Albiate in forma – a project promoting a healthier lifestyle and habits" from Italy (http://www.piedibus.it/upl/biblioteca/1152783714_ALBIATE%20IN%20FORMA.pdf, accessed 21 December 2010);
   - "Bike It – a school cycling project" from the United Kingdom (http://www.sustrans.org.uk/what-we-do/bike-it, accessed 21 December 2010);
   - "Community Food Cooperatives – a project to supply fruit and vegetables from locally produced sources" from Wales, United Kingdom (http://www.physicalactivityandnutritionwales.org.uk/page.cfm?orgid=740&pid=29570, accessed 21 December 2010);
   - "EPODE France – Together let's prevent childhood obesity – a community-based intervention to prevent childhood obesity with local stakeholders" implemented in various European countries (http://www.epode.org/, accessed 21 December 2010);
   - "Happy Body – a project to enhance fitness of the Belgian population via the promotion of healthy nutrition and physical activity" from Belgium (http://www.happybodytoyou.be/, accessed 21 December 2010);
   - "Healthy School Canteen – a programme to establish healthy school canteens in secondary schools" from the Netherlands (http://www.degezondeschoolkantine.nl, accessed 21 December 2010); and
   - "Programme on nutrition prevention and health of children adolescents in Aquitaine – a programme that was initiated to stabilize the prevalence of childhood obesity" (http://www.nutritionenfantaquitaine.fr/, accessed 21 December 2010).
4. The appraisal form (second component of the tool) was pilot tested by various experts, who were asked to independently appraise one of the seven programmes and to make comments on the tool.

The tool components

The tool consists of three parts.

1. **The questionnaire** serves as the information-gathering form for the tool. Programme managers are asked to answer 43 questions and provide relevant reference materials, such as a programme description, internet links, evaluation report, overview of budget and time-line. The questionnaire comprises the following three sections.
   - *Main intervention characteristics.* This consists of questions related to the general design and planning of a programme, such as the main objectives, planned activities, target group and involved stakeholders.
   - *Monitoring and evaluation.* This consists of questions related to the monitoring and evaluation process and thus addresses indicators, statistics and measurements.
   - *Implementation.* This consists of questions related to the implementation stage of the intervention and refers to performance, programme management and target group participation.

2. **The appraisal** form, with 43 criteria statements, serves as a check list for reviewers to assess the information gathered in the questionnaire.

3. **A scoring** sheet allows one to calculate a good practice score for each of the three sections as well as for the programme as a whole.

Scoring of good practice

An indication of good practice is obtained for each section as well as for the intervention as a whole. This makes it possible to highlight programmes that may, for example, have a very good design but poor evaluation and implementation, or programmes that are well-evaluated but struggle with design and implementation, or programmes that are not well-designed and evaluated but nevertheless have an excellent implementation. For ongoing programmes, only the first section of the questionnaire and appraisal form can be completed.

First, a total score is obtained for each section. This is divided by the maximum section score, leading to section scores less than or equal to unity. A score of 0.8 or higher in a section certifies a programme as "good practice" in the respective section, a score of 0.6–0.8 refers to acceptable practice, a score of 0.4–0.6 indicates marginal practice and a score below 0.4 refers to weak practice. Then, based on the outcome of the three section scores, an average good practice score for the programme is calculated.

For the calculation of the scores, a distinction is made between core questions and general questions. A higher weighting is given to core questions than to general questions, as these are considered to be more crucial in quality assessment. Core questions are therefore multiplied by a factor of 3 and general questions are given one mark.

Assessment of programmes

The Regional Office will apply the following steps for the assessment of good practice elements of public health programmes that aim to improve nutrition and physical activity or prevent obesity in the general population.

Step 1. Completion of questionnaire by coordinator. The programme coordinator is asked to answer the 43 questions of the questionnaire and provide relevant reference materials, such as a programme description, internet links, evaluation report, overview of budget and time-line. After completion, the coordinator is requested to send the questionnaire back to the Regional Office.

Step 2. Assessment of good practice using the appraisal form. The Regional Office has established a roster of experts to assist in the appraisal of programmes. Each programme will be reviewed independently by two of these experts. For each programme, experts will be asked to complete the appraisal form, depending on their expertise or area of work within the programme to be appraised.

Step 3. Scoring. On the basis of the two completed appraisal forms, a good practice score is calculated for each section as well as for the whole programme.

Step 4. Inclusion in database. A description of the programme and the obtained score are incorporated into the NOPA database (http://data.euro.who.int/nopa, accessed 19 May 2011).

Questionnaire to gather information on obesity prevention programmes

Instructions

We kindly ask the coordinator to complete the questionnaire and provide any relevant reference material/programme documentation, e.g. general programme description, report on the outcomes, programme evaluation, scientific publications, links to web sites, etc.

The information provided will be treated confidentially. Only final scores from each section, the programme description provided by you and information from marked areas will be included in the database.

You may need to consult your colleagues before completing certain questions.

General programme information

✓ Name of the programme:

✓ Country and region (if applicable) where the programme is based:

✓ Web site (if existing):

✓ Time period (start and end dates):

✓ Funding sources:

✓ Time period covered by the funding:

✓ Total budget:

✓ Number of staff (both paid and unpaid) involved:

✓ Give a short description of the programme (maximum of about 300 words):

Contact details of programme

✓ Name and job title:

✓ Organization:

✓ E-mail address:

✓ Postal address and telephone number:

I. Main intervention characteristics

1. Describe the overall aim(s) of the intervention.

2. Indicate which of the following components are addressed by the intervention.

☐ Healthy eating
☐ Physical activity
☐ Other

Please specify:

3. List the objectives of the intervention.

4. Is the intervention based on current scientific knowledge and/or theoretical models and/or previous experience from other projects?

- [ ] Yes, current scientific knowledge
- [ ] Yes, current theoretical models
- [ ] Yes, previous experience
- [ ] No

Please provide further details about your answer option:

5. Were existing (inter)national diet and physical activity guidelines taken into account during the development of the intervention?

- [ ] Yes

Please specify the guidelines, the publisher and the publication date:

- [ ] No

Please explain why not:

6. Has a needs assessment been carried out?

- [ ] Yes

Please specify the results of the needs assessment:

- [ ] No

Please explain why not:

7. Describe the planned key activities.

8. Does the intervention also address environmental factors (i.e. factors beyond individual control)?

- [ ] Yes

Please specify which factors are addressed and how:

- [ ] No

Please explain why not:

9. Is the approach of the intervention designed to have a lasting effect on the risk factors?

☐ Yes

Please provide further details:

☐ No

Please explain why not:

10. Describe the structures within which the intervention was carried out.

☐ Existing structures (e.g. part of the administration, nongovernmental organization, etc.)
☐ Newly created structure that will continue to exist after the intervention is concluded
☐ Newly created structure that will not continue to exist after the intervention is concluded
☐ No specific structure (e.g. project team)

Please provide further details about the indicated answer option:

11. Describe the target group(s) of the intervention.

12. Does the intervention have a special focus on vulnerable groups (socioeconomically disadvantaged people, ethnic minorities, children, elderly people, etc.)?

☐ Yes

Please specify the vulnerable groups:

☐ No

Please explain why not:

13. Does the intervention aim to empower the target group(s)?

☐ Yes

Please specify:

☐ No

Please explain why not:

14. Was/were the target group(s) involved in setting the objectives and designing the intervention?

☐ Yes

Please specify:

☐ No

Please explain why not:

15. Have possible adverse effects of the intervention on the target group(s) been considered and minimized?

Explanation: An adverse effect is a harmful and undesired effect resulting from an intervention.

☐ Yes

Please specify:

☐ No

Please explain why not:

16. Describe the involvement of stakeholders in the planning phase of the intervention and specify the stakeholders.

Explanation: a stakeholder is a person, group or organization that affects or can be affected by the intervention.

17. Specify the sectors represented by the professionals that were involved in the intervention (e.g. health, transport, environment, education, etc.) and describe their role in the intervention.

18. How much of the total budget was allocated to the evaluation of the programme (as a percentage of the total budget)?

19. How was the programme management carried out?

☐ A timetable in which tasks, activities and responsibilities were clearly described
☐ Day-to-day-planning with programme team
☐ Other technique, namely: ______
☐ No specific programme management technique was applied

Please provide further details about your answer option:

II. Monitoring and Evaluation

20. Has resource utilization (funds, human resources, materials) for the intervention been monitored?

☐ Yes

Please specify the indicators and their frequency of measurement:

☐ No

Please explain why not:

21. Describe how the process of the intervention was measured.

☐ Specific indicators were used

Please specify the indicators and the frequency of measurement for each indicator:

☐ Summary evaluation was carried out at the end of the intervention

☐ No specific monitoring or evaluation was carried out

Process indicators are used to measure progress in the processes of change and to investigate how something has been done, rather than what has happened as a result. An example is the setting up of an expert advisory committee with active responsibility for quality assurance of the intervention or adherence to the time plan of the programme. Process indicators should be measurable (use at least qualitative dimensions), factual (mean the same to everyone), valid (measure what they claim to measure), verifiable (be able to be checked) and sensitive (reflect changes in the situation).

22. Describe how the output of the intervention was measured.

☐ Specific indicators were used

Please specify the indicators and the frequency of measurement for each indicator:

☐ Summary evaluation was carried out at the end of the intervention

☐ No specific monitoring or evaluation was carried out

Output indicators are used to quantify conducted activities, for example the total number of participants. They are also used to measure the outputs or products that result from processes, such as the publication of a booklet on healthy diets. Output indicators can also include improving the social and physical environments of various settings to support the adoption of healthier types of behaviour, such as improved access to fruit and vegetables or safe cycling routes. They should be linked to the objectives and be measurable, factual, valid, verifiable and sensitive.

23. Describe how the outcome of the intervention was measured.

- [ ] Specific indicators were used

Please specify the indicators and the frequency of measurement for each indicator:

- [ ] Summary evaluation was carried out at the end of the intervention
- [ ] No specific monitoring or evaluation was carried out

Outcome indicators are used to measure the ultimate outcomes of an action. Depending on the specified objectives, these might be short-term (such as increased knowledge), intermediate (such as change in behaviour) or long-term (such as reduction in incidence of cardiovascular disease). An example is the reduction of the percentage of primary school children in the community of Sandes not reaching the minimum recommended amount of physical activity by 5%. They should be related to the targets as well as quantifiable, factual, valid and verifiable.

24. Indicate the demographic and socioeconomic factors of the target population that have been measured.

- [ ] Age
- [ ] Gender
- [ ] Income/socioeconomic status
- [ ] Education
- [ ] Occupation
- [ ] Ethnicity
- [ ] Geographical location
- [ ] Other, namely:

25. Was a long-term follow-up carried out after the end of the intervention?

- [ ] Yes

Please specify how many months after the end of the intervention:

- [ ] No

Please explain why and continue with question 27:

26. Describe the sample of the study population that was monitored as part of the follow up *(please give a percentage)*.

27. Were statistical methods used in the evaluation of the intervention?

- [ ] Yes

Please specify:

- [ ] No

Please explain why not:

28. Were confounding factors taken into consideration?

Explanation: A confounding factor is a variable that can cause or prevent the outcome of interest, is not an intermediate variable, and is associated with the factor under investigation. A confounding factor may be due to chance or bias. Unless it is possible to adjust for confounding variables, their effects cannot be distinguished from those of factor(s) being studied.

☐ Yes

Please specify:

______

______

______

☐ No

Please explain why not:

______

______

______

29. Have cost–effectiveness calculations been made?

Explanation: Cost–effectiveness compares the relative expenditure (costs) and outcomes (effects) of two or more courses of action. Typically cost–effectiveness is expressed in terms of a ratio, where the denominator is a gain in health from a measure (e.g. years of life, sight-years gained) and the numerator is the cost of the health gain. A special case is cost–utility analysis, where the effects are measured in terms of years of healthy life lived, using a measure such as quality-adjusted life years (QALY) or disability-adjusted life years (DALY).

☐ Yes

Please provide further details about how the calculations were made:

______

______

______

☐ No

Please explain why not:

______

______

______

30. Has an evaluation of the intervention been carried out?

☐ Yes, an external evaluation
☐ Yes, an internal evaluation
☐ Yes, both internal and external evaluations
☐ No (*please go to part III*)

Please provide further details about the evaluation that has been carried out:

______

______

______

31. Are stakeholders' opinions assessed in monitoring and evaluation?

☐ Yes

Please specify and indicate the respective stakeholders:

☐ No

Please explain why not:

32. Is the monitoring and evaluation process described in the main programme documentation?

☐ Yes

Please give an overview and provide a reference:

☐ No

Please explain why not:

III. Implementation

33. Has a pilot study been performed?

☐ Yes

Please provide details of the pilot study:

☐ No

Please explain why not:

34. Describe the activities that have been carried out.

35. Describe the performance of the intervention in terms of time management and the activities that were undertaken to ensure high-quality delivery.

36. Describe which stakeholders were involved in the implementation and describe their roles.

37. Is the initiative coordinated or linked with other relevant interventions?

☐ Yes

Please specify the intervention(s):

☐ No

Please explain why not:

38. Provide an overview of the resources that were invested and indicate where more information can be found.

39. Is the implementation process described in the main programme documentation?

☐ Yes

Please give an overview and provide a reference:

☐ No

Please explain why not:

40. Has actual outcome performance been measured against a control group?

☐ Yes

Please specify where further documentation on the outcome performance can be found:

☐ No

Please explain why not:

41. Has the planned target group participation been reached?

☐ Yes

Please specify:

☐ No

Please explain why not:

42. To what extent have the planned key activities indicated in section I (question 7) been carried out? *(Please give a percentage):*

43. To what extent have the objectives indicated in section I (question 3) been achieved? *(Please give a percentage):*

Appraisal form – a checklist for reviewers

I. Main intervention characteristics

Targets

1. The aims of the intervention are clearly described.

- [ ] Yes
- [ ] No

2. The intervention combines healthy eating and physical activity.

- [ ] Yes
- [ ] No

3. SMART objectives are provided.

- [ ] Yes, at least 3 of the 5
- [ ] No, or not specified

Explanation: SMART objectives are:

1. ***S**pecific: objectives should clearly specify what is to be achieved*
2. ***M**easurable: objectives should be phrased in a way that achievement can be measured*
3. ***A**chievable: objectives should refer to something that the intervention can actually influence and change*
4. ***R**ealistic: objectives should be realistically attainable within the given time frame and with the available resources (human and financial resources and capacity)*
5. ***T**ime-bound: objectives should relate to a clearly stated time frame.*

Relevance

4. The intervention is based on current scientific knowledge and/or theoretical models and/or previous experience.

- [ ] Yes, current scientific knowledge
- [ ] Yes, theoretical models
- [ ] Yes, previous experience
- [ ] No, or not specified

5. The intervention acts in coherence with existing diet and/or physical activity guidelines.

- [ ] Yes, the intervention acts in coherence with national or international guidelines
- [ ] No, or other guidelines, or not specified

6. A needs assessment has been performed.

- [ ] Yes
- [ ] No, or not specified

7. Planned key activities are relevant to the needs of the target group.

- [ ] Yes
- [ ] No, or not specified

8. The activities also address environmental factors (i.e. factors beyond individual control).

- [ ] Yes
- [ ] No, or not specified

Examples
School: provision of healthy meals in the canteen, school fruit and vegetable schemes, removal or change of contents of vending machines, provision of cheap or free water supply.
Workplace: promotion of stair use, availability of facilities for physical activity and showers for staff coming by bicycle, provision of healthy meals in the canteen, promotion of participation in sports, such as a company marathon team.
Community: improved information and access to a choice of healthier foods and to sport and recreational facilities and green spaces for physical activity, availability and accessibility of a safe transport infrastructure and of institutional or organizational incentives for non-motorized means of transportation, presence of aesthetic attractions and comforts as well as absence of physical disorder.
Media: improved image of healthy eating and living through in television, video games and billboards.

Sustainability

9. The intervention is designed to have a lasting effect on the risk factors.

- [ ] Yes
- [ ] No, or not specified

10. The activities are taking place within structures that can carry on the intervention.

- [ ] Yes
- [ ] No, or not specified

Examples

School: inclusion of nutrition education in the curriculum, teacher training in the promotion of healthy nutrition and/or physical activity.
Workplace: presence of staff canteens serving quality meals, provision of facilities for physical activity in the workplace (e.g. gym, basketball court).
Transport: improved provision of walking and cycling routes, promotion of stair use in public buildings.
Community: provision of information on nutrition in local stores, improvement of the aesthetics of the environment.
Media: popular soap operas promote healthy choices and active living.

Target group

11. The target group(s) is/are clearly stated.

- [ ] Yes
- [ ] No, or not specified

12. There is a special focus on vulnerable groups (socioeconomically disadvantaged people, ethnic minorities, children, elderly people, etc.).

- [ ] Yes
- [ ] No, or not specified

13. The intervention aims to empower the target group(s).

- [ ] Yes
- [ ] No, or not specified

Explanation: *The intervention increases the capacity of individuals or groups to make choices about their health and to transform those choices into desired actions and outcomes by strengthening personal abilities such as self control, confidence and autonomy.*

14. The target group(s) has/have been involved in setting the objectives and designing the intervention.

- [ ] Yes
- [ ] No, or not specified

15. Possible adverse effects of the intervention were considered and minimized.

- [ ] Yes
- [ ] No, or not specified

Partners and cooperation

16. The main stakeholders were involved in the planning phase of the intervention.

- [ ] Yes, all
- [ ] Yes, at least one
- [ ] No, or not specified

Examples of stakeholders

Family and preschool: parents, social workers, kindergarten or nursery teachers, children.
School: children, parents, teachers, school board members, food providers.
Workplace: employees, company board members, staff association, food providers.
Community: community members, community board members, social workers of ongoing projects or established institutions.
Media: target group members, advocacy groups of the target group (such as representing youth, ethnic groups, women, socioeconomically disadvantaged people), experts in this field of action, governing health policy department.

17. The intervention involves professionals from different sectors.

- [ ] Yes
- [ ] No, or not specified

Planning

18. A proportion of the budget is allocated to monitoring and evaluation.

- [ ] Yes, 5% or more
- [ ] Yes, less than 5%
- [ ] No, or not specified

19. A timetable has been set in which tasks, activities and responsibilities are clearly described.

☐ Yes
☐ No, or not specified

II. Monitoring and evaluation

Indicators and monitoring

20. Resource utilization (funds, human resources, materials) have been monitored.

☐ Yes
☐ No, or not specified

21. Process indicators are measured regularly.

☐ Yes
☐ No, or not specified

Explanation: Process indicators are used to measure progress in the processes of change and to investigate how something has been done, rather than what has happened as a result. An example is the setting up of an expert advisory committee with active responsibility for quality assurance of the intervention or adherence to the time plan of the programme. Process indicators should be measurable (use at least qualitative dimensions), factual (mean the same to everyone), valid (measure what they claim to measure), verifiable (be able to be checked) and sensitive (reflect changes in the situation).

22. Output indicators are measured regularly.

☐ Yes
☐ No, or not specified

Explanation: Output indicators are used to quantify conducted activities such as the total number of participants. They are also used to measure the outputs or products that come about as the result of processes, for example the publication of a booklet on healthy diets. Output indicators can also include improving the social and physical environments of various settings to support the adoption of healthier types of behaviour, such as improved access to fruit and vegetables or safe cycling routes. They should be linked to the objectives and be measurable, factual, valid, verifiable and sensitive.

23. Outcome indicators are measured regularly.

☐ Yes
☐ No, or not specified

Explanation: Outcome indicators are used to measure the ultimate outcomes of an action. Depending on the specified objectives, these might be short-term (such as increased knowledge), intermediate (such as change in behaviour) or long-term (such as reduction in incidence of cardiovascular disease). An example is the reduction of the percentage of primary school children in the community of Sandes not reaching the minimum recommended amount of physical activity by 5%. They should be related to the targets as well as quantifiable, factual, valid and verifiable.

Measurements

24. Demographic and socioeconomic factors of the target population are measured (age, gender, income/socioeconomic status/education, occupation, ethnicity and geographical location).

☐ Yes, at least one of the above-mentioned factors
☐ No

25. A long-term follow-up was performed at least 6–12 months after the intervention.

☐ Yes
☐ No, or not specified

26. The follow-up is performed in a representative sample of the target group and includes more than 80% of the evaluation sample.

☐ Yes
☐ No, or not specified

Statistical methods

27. The statistical methods are described.

☐ Yes
☐ No, or not specified

28. Confounding factors are taken into consideration.

☐ Yes
☐ No, or not specified

Explanation: The theme of confounding is mentioned and existing confounding factors are explained (if reported) and the extent of confounding is discussed.

Result assessment

29. Cost–effectiveness calculations are made.

☐ Yes
☐ No, or not specified

Explanation: Cost–effectiveness compares the relative expenditure (costs) and outcomes (effects) of two or more courses of action. Typically cost–effectiveness is expressed in terms of a ratio, where the denominator is a gain in health from a measure (e.g. years of life, sight-years gained) and the numerator is the cost of the health gain. A special case is cost–utility analysis, where the effects are measured in terms of years of healthy life lived, using a measure such as quality-adjusted life years (QALY)or disability-adjusted life years (DALY).

30. External and/or internal evaluations have been performed.

☐ Yes, both
☐ Yes, an external evaluation
☐ Yes, an internal evaluation
☐ No, or not specified

Stakeholders

31. Stakeholders' opinions are assessed in monitoring and evaluation.

☐ Yes
☐ No, or not specified

Communication

32. The monitoring and evaluation process is described in the main intervention documentation.

☐ Yes
☐ No, or not specified

III. Implementation

Performance

33. A pilot study has been performed.

☐ Yes
☐ No, or not specified

34. The activities that are carried out are relevant to the objectives of the intervention (compare with question 3 under main intervention characteristics).

☐ Yes, all
☐ Yes, partially
☐ No, or not specified

35. The intervention was implemented according to the timetable, and activities to ensure high-quality delivery were carried out.

☐ Yes
☐ No, or not specified

Partners and cooperation

36. Relevant stakeholders are involved in the implementation.

☐ Yes, all
☐ Yes, at least one
☐ No, or not specified

Examples
Family and preschool: parents, social workers, kindergarten or nursery teachers, children.
School: children, parents, teachers, school board members, food providers.
Workplace: employees, company board members, staff association, food providers.
Community: community members, community board members, social workers of ongoing projects or established institutions.
Media: target group members, advocacy groups of the target group (such as representing youth, ethnic groups, women, socioeconomically disadvantaged people), experts in this field of action, governing health policy department.

37. The initiative is coordinated and linked with other relevant interventions.

☐ Yes
☐ No, or not specified

Explanation: Networking can strengthen the sustainability of the programme and is an indicator of transparency and willingness to learn from others.

Communication and documentation

38. Resource information (funds, human resources, materials) is described in the main programme documentation.

☐ Yes
☐ No, or not specified

39. The implementation process (activities, staff affiliations, timetable, monitoring and evaluation) is described in the main programme documentation.

☐ Yes
☐ No, or not specified

40. The main programme documentation is publicly accessible (a web link is provided).

☐ Yes
☐ No, or not specified

Target group participation

41. The planned target group participation has been reached.

☐ Yes
☐ No, or not specified

Achievement of intervention objectives

42. A minimum of 70% of planned activities have been performed.

☐ Yes
☐ No, or not specified

43. At least 90% of the objectives have been achieved.

☐ Yes
☐ No, or not specified

References

1. Branca F, Nikogosian H, Lobstein T, eds. *The challenge of obesity in the WHO European Region and the strategies for response*. Copenhagen, WHO Regional Office for Europe, 2007 (http://www.euro.who.int/__data/assets/pdf_file/0010/74746/E90711.pdf, accessed 21 December 2010).
2. *Report of the meeting on community initiatives to improve nutrition and physical activity. Berlin, Germany, 21–22 February 2008*. Copenhagen, WHO Regional Office for Europe, 2010 (http://ec.europa.eu/health/nutrition_physical_activity/docs/implementation_report_a1c_en.pdf, accessed 21 December 2010).
3. Molleman GRM et al. Project quality rating by experts and practitioners: experience with Preffi 2.0 as a quality assessment instrument. *Health Education Research, Theory and Practice*, 2006, 21:219–229.
4. Molleman G et al. *Health Promotion Effect Management Instrument Preffi 2.0: assessment package*. Woerden, Netherlands Institute for Health Promotion and Disease Prevention, 2003 (http://www.nigz.nl/inc/pdf.cfm?pad=misc&pdf=Assesment_package_Preffi_211.pdf, accessed 21 December 2010).
5. Peters L et al. *Explanatory Guide Preffi 2.0*. Woerden, Netherlands Institute for Health Promotion and Disease Prevention, 2003 (http://w3.nigz.nl/inc/getdocument.cfm?filename=upload/Preffi%202_0_%20Explanatory_Guide.pdf, accessed 21 December 2010).
6. *A survey of Australia's community-based obesity prevention projects*. Geelong, CO-OPS Secretariat, Deakin University, 2009.
7. King L, Gill T. *Best Practice. Principles for community-based obesity prevention*. Geelong, CO-OPS Secretariat, Deakin University, 2009.
8. Getting Evidence into Practice Project, Consortium. *European Quality Instrument for Health Promotion (EQUIHP)*. Netherlands Institute for Health Promotion and Disease Prevention, Flemish Institute for Health Promotion, 2005 (http://ws5.e-vision.nl/systeem3/images/Annexe%2010%20EQUIHP.pdf, accessed 21 December 2010).
9. Bollars C et al for the Project Getting Evidence into Practice Project Consortium. *User Manual: European Quality Instrument for Health Promotion (EQUIHP).* Netherlands Institute for Health Promotion and Disease Prevention, Flemish Institute for Health Promotion, 2005. (http://ws5.e-vision.nl/systeem3/images/User%20Manual1.pdf, accessed 21 December 2010).
10. McNeil DA, Flynn MAT. Methods of defining best practice for population health approaches with obesity prevention as an example. *Proceedings of the Nutrition Society*, 2006, 65:403–411.
11. Leemrijse CJ et al. *Evaluating promising interventions to stimulate physical activity in the population*. Utrecht, Netherlands Institute for Health Services Research, 2009 (http://www.nivel.nl/pdf/Rapport-evaluatie-kansrijke-beweegprogrammas-fase2.pdf, accessed 21 December 2010).
12. Scheurs MHGM et al. Tool for evaluating initiatives of private food sector to generate best practices in reducing overweight. *Annals of Nutrition and Metabolism*, 2007, 51(Suppl. 1):320.
13. Swinburn B et al. Obesity prevention: a proposed framework for translating evidence into action. *Obesity Reviews*, 2005, 6:23–33.
14. *Intervention database for health promotion and prevention "Loketgezondleven"*. Bilthoven, Dutch National Institute for Public Health and the Environment, 2007 (http://www.loketgezondleven.nl/interventies/i-database/, accessed 21 December 2010).
15. Koplan JP et al., eds. *Progress in preventing childhood obesity: How do we measure up?* Washington, DC, National Academies Press, 2007 (http://www.nap.edu/openbook.php?record_id=11722, accessed 21 December 2010).

For further information please contact:

Noncommunicable Diseases and Health Promotion
Nutrition, Physical Activity and Obesity:

Trudy Wijnhoven
Technical Officer, Nutrition Surveillance
E-mail: twi@euro.who.int

World Health Organization
Regional Office for Europe
Scherfigsvej 8, DK-2100 Copenhagen Ø, Denmark
Tel.: +45 39 17 17 17. Fax: +45 39 17 18 18.
E-mail: postmaster@euro.who.int
Web site: www.euro.who.int
