# Supplementary material for: Tools for a systematic appraisal of integrated community-based approaches to prevent childhood obesity
Source: BMC Public Health. 2018 Jan 29;18:189. doi: 10.1186/s12889-018-5042-4 (PMC5789618; doi:10.1186/s12889-018-5042-4)
Supplement: Supplementary file 2 — OPEN tool question list. (DOCX 37 kb) [file 12889_2018_5042_MOESM2_ESM.docx]

**Additional file 2.** OPEN tool question list

Name:

Position:

Name of the programme:

City-Country**:**

**Questions for the main programme coordinator**

*The aim of this interview is to better understand your organization and your childhood obesity prevention activities in order to provide you with practical advice, based on the experience we have in implementing community-based programmes. We ask you to give your point of view on the following themes: general organization, political involvement, public & private partnership, campaigns & interventions, communication, scientific aspects and budget.*

| **A. General –How is the programme organised** |
| --- |

1. What organization (government, company, NGO…) and what department are you part of?

2. What is your background?

1. Have you followed any extra training for this programme? Please describe.

3. How long have you been working on the XXX programme?

4. How many days/hours per week do you work on the XXX programme?

5. Has a team been organised as a result of the xxxx programme?

a. How is this team organized?

i. How many members working for the programme

ii. Their expertise

iii. Their task/duties (in the programme)?

6a. Are there on-going programmes / campaigns as part of a National Plan on obesity prevention / promotion of healthy lifestyle?

6b. Does your programme fit in that plan?

• How?

7. Please describe the communities reached and target groups approached

1. Number of communities
2. Number of people
3. Age groups

| **B. Political involvement at National/Regional level** |
| --- |

1. Is there a formal agreement to the programme at National/regional level with political structure?

-If yes,

1. What does it include?

- Tasks/responsibilities of the parties? What do(es) political partner(s) provide to the XXX programme (e.g. Financial ? / Expertise? / Benefits in kind: physical space, communication materials, manpower, evaluation, data use?)?
- How do they contribute?

1. Who is responsible in y our programme /team for ensuring political commitment at national*/local* level?

*2a. Is the programme supported within the municipality?*

*2b. How does this translate in practice (structures, organisations, human resources, advocacy, funds)*

3. Do political partners actively advocate the programme? How?

4. What do the National/Regional political representatives think about the programme? (supporters, neutral, against)

5. Are you working directly with a political representative representing the programme?

1. Who

*6. Do the other sectors of the municipality contribute to the XXX programme?*

1. *How? Is there cooperation between these sectors (inter-sectoral cooperation)?*
2. *Which ones?*
3. *Do elected representatives of the municipality contribute to the programme?*

7. How do you communicate the progress of the programme to your political partners? Telephone calls – frequency; Emails – frequency ;Reports – frequency

1. Meetings – frequency

8. When are the next National/Regional elections?

9a. Are you satisfied with the established political commitment?

9b. What is needed to progress?

| **C. Public-private partnership** |
| --- |

1. Are there public/private partnerships involved?

-If yes,

a. Who is responsible for creating/handling the PPPs in your team? What are his/her tasks regarding it?

b. How PPP is being applied in the national/regional level? What does it mean for the programme (parties involved in the agreement – NGO and communication agency, national/local authorities and private partners, government and NGO/for-profit organisation-, how important is it for the programme to have an agreement between these parties?

c. How is this activity (e.g. time/materials/personnel spent for recruitment and management) financed?

d. Helping factors/barriers for the development of PPP?

-If no,

- Why not?
- Do you plan to involve a partnership like this in xxxx? In what way?
- Who, why, when?

2. Has knowledge been acquired for the development and management of PPP, and if so how?

- Existed expertise or training(s)? Could you explain?
- If training, by whom? Was it helpful?
- Do you use it in your work? If yes, how?

3. What is the position of the National/Regional government regarding PPP?

4. What is the

1. Society’s
2. Scientific community’s view on PPP?

5. Who are your actual partners?

1. public
2. private
3. Are there any potential partners?
   - 1. Public
     2. private

List to ask:

| Government:  Universities:  Hospitals:  Health-others:  Associations: | Media  Companies:  Foundations:  Religious bodies:  Political bodies: |
| --- | --- |

6. Do you have a PPP charter?

- Is it programme-specific or a pre-existing document? Which one?
- Have you undergone conflict of interest issues? How did you solve the problem?

7. How do these parties contribute to the programme?

- Financial? / Expertise? / Benefits in kind: physical space, communication materials, manpower, evaluation, data use?

1. Public partners
2. Private partners
   - How is this agreed? (price + period)

8. Why did these partners join the programme?

a. How did you convince them?

b. What is the advantage for them to join the programme? / Good reputation? / Health of employees? / corporate social responsibility / other)?

c. How do you keep them motivated (regular meetings, annual reviews, media coverage reports, evaluation reports, etc.)?

9. How often do you meet your private partners and on what occasions (single meetings, events etc.)

10. Are you satisfied with the established PPP?

1. What is needed to progress?

| **D. Development of interventions and campaigns at National/Regional level** |
| --- |

1. Do you develop and implement interventions and/or campaigns? Specify

1. At what level?

2. Has knowledge been acquired regarding the design and deployment of interventions and/or campaigns? specify

-if so

1. Existed expertise or training(s)? Could you explain?
2. If training, by who and was it helpful?
3. Do you use it in your work and how?

3. Which are the target groups of your interventions / campaigns? Specify

1. Final target group (s)
2. Parent as target group
3. Other intermediate target group (s) (e.g. local managers)
4. Do you consider the local stakeholders participating in the interventions and campaigns as target groups?

4. Has a target group analysis been done? If yes, how did you use it?

5. Could you please describe the process of the **planning** of an intervention?

a. Who is responsible for this? What are his/her tasks?

b. How is the theme decided? On what basis (needs analysis, focus groups, ITVs, feedback from local project managers). Please give details of the process.

c. Who else is involved and what are the specific tasks?

1. Is the final target group involved in the planning phase?
2. Any private partners?

d. Are you satisfied with the overall process for **planning** the interventions and campaigns?

1. What is needed to progress?

6. Could you please describe the process of the **implementation** of an intervention?

a. Who is responsible for this? What are his/her tasks?

b. Who is developing the tools? Form contents to graphic design and printing.

c. Is there anyone validating the contents of the tools/intervention? Please give details

d. Who else is involved and what are the specific tasks. Private partners?

e. Is there any training of the people involved in the field (form LPM to volunteers)?

f. Are you satisfied with the overall process for **implementing** the interventions and campaigns?

1. What is needed to progress?

7. What tools do you develop for your target groups? (e.g. intervention guide, poster, leaflet, book, recipe sheet)

8. If there are on-going programmes / campaigns on childhood obesity prevention or on other related public health issues, do you make use of their tools? How?

9. Do you use the experience of the local coordination teams/ actors directly involved for the design of future interventions/campaigns?

- If yes, how (the actions they have implemented, their methodology, the tools they have developed, their feedback on the tools etc)?
- If not, why?

10. What do your interventions/ campaigns (specify) include in terms of:

1. Themes: Food habits/physical activity/ other
   1. (micro/macro)environmental change/
2. Activities: organization of events, workshops, PR/ other

11. Can you describe the last intervention/campaign (specify) you have led?

• Duration

• When was it

• Theme

• Methodological tools

• Communication tools

• Intervention tools

• Was it was a success/not a success? Why

Can you provide us with some materials?

*Local level:*

- *Do you include other existing actions in the town in the XXX programme? Which are they?*
- *To what extend do you use the materials (methodological, communication, intervention tools) provided by the national coordination? Why?*

| **E. Communication on the programme at National/Regional level** |
| --- |

1. Has knowledge been acquired on communication / PR activities?

a. Existing expertise or training(s)? Could you explain?

b. If trainings, from where? was it helpful?

a. Do you use it in your work and how?

2. What communication materials and channels do you use*?

*Website, Newsletter, Press releases, Press events, One-to-one direct communication (journalists, political, scientific, private partners), Newspapers, TV, social media, Web TV, Radio, Facebook, Twitter, Google +, Google groups

3. Who is responsible for the communication of the programme?

- Internal/external?
- Why is it organized like this? (if not answered previously)
- Does it work well?
- Helping factors/barriers?

4. Are your PPP partners involved in the PR communication activities?

- If yes, how?

- Advantages – disadvantages of this involvement?

5. Is there a communication plan?

- If yes, how often is it being set-up?

6. Do you evaluate your PR communication?

a. What are your results in terms of media coverage/visibility?

b. Are you satisfied with these results? Why?

| **F. Scientific aspects at National/Regional level** |
| --- |

1. Is scientific support used within the programme?

a. Did you create a scientific advisory board or individual experts are collaborating occasionally, or both? Please explain.

b. Who is part of it? What are their areas of expertise?

c. What are their responsibilities within the programme? (tools contents, validation, evaluation, publication, spokesperson)

d. Do you have scientific spokespersons?

- Who?

e. How often do you meet the scientific experts and for what purpose?

f. How is it financed?

g. How do you feel about this collaboration?

- Does it work well?
- Why?

| 2. Have knowledge been acquired on the scientific aspects of the programme (understanding obesity, intervention protocol, evaluation) and if so how?     - Do you use it in your work? How? |
| --- |
|  |
| 3. Does an evaluation take place?   1. Is there an evaluation framework?    1. If yes, Who developed it? Does it follow a specific methodology (e.g a logic model, definition of SMART objectives)? 2. Who is responsible for the evaluation?   4. What is evaluated?   1. Processes:    1. Central coordination    2. Local coordination    3. Setting/actions 2. Effects:    1. Behavioral change of children/ families    2. BMI   **If the interviewee explains in detail, make sure the elements in q5 are mentioned.** |
| 5. At what points does evaluation take place?   - - Why is it organised like this?   - How is this financed? - Does it work well? Why? |
| 6. Are you satisfied with the evaluation process of your programme (what is evaluated, funding, feasibility)?   1. What is needed to progress?   7. Do you have implementation and evaluation results?   1. How do you use them (scientific publication, dissemination, other use) 2. Do you have scientific publications?   COLLECT PUBLICATIONS  8. What scientific events do you participate in and how (poster, presentation, workshop…)? |
| 9. What scientific events do you attend?  10. Budget (% or €) for evaluation activities |

**Footnotes**

^: Questions addressed to the intervention level
