# Supplementary material for: Tools for a systematic appraisal of integrated community-based approaches to prevent childhood obesity
Source: BMC Public Health. 2018 Jan 29;18:189. doi: 10.1186/s12889-018-5042-4 (PMC5789618; doi:10.1186/s12889-018-5042-4)
Supplement: Supplementary file 3 — Analysis grid of the OPEN tool. (DOCX 56 kb) [file 12889_2018_5042_MOESM3_ESM.docx]

**Additional file 3**. Analysis grid of the OPEN tool.

| **A. GENERAL CHARACTERISTICS OF THE PROGRAMME** | | | | | | | | | | |
| --- | --- | --- | --- | --- | --- | --- | --- | --- | --- | --- |
| **CODE** | **ELEMENT** | **INTERVIEW QUESTION** | | **ANSWER** | **SCORING CATEGORIES** | **REASERCHERS’ COMMENT** | | **POINTS SCORED** | | **MAX POINTS** |
| **A1** | **Structure** | What organization (government, company, NGO…) and what department are you part of? | |  |  | Descriptive | | NOT SCORED | | |
| **A2** | **Background** | What is your background? a. Have you followed any extra training for this programme? Please describe. | |  |  |  | | NOT SCORED | | |
| **A3** | **How long working for the programme** |  | |  |  |  | | NOT SCORED | | |
| **A4i** | **Principal programme Coordinator Commitment** | How many days/hours per week do you work on the XXX programme? | |  | 1. Part time, some full time 2. Full time | The ones operating at the same level and share the tasks are combined. | |  | | 2 |
| **A4ii** | **Project Coordinator Commitment (local level)** |  | |  | 1. Part time, some full time 2. Full time | The ones operating at the same level and share the tasks are combined. | |  | | 2 |
| **A**5i | **Programme team** | Has a team been organised as a result of the programme?  ai. How many members working for the programme | |  | 0. 1 person 1. 2-3 people 2. more than 3 |  | |  | | 2 |
| **A5ii** | **Programme team expertise** | aii. Their expertise | |  | 0. One expertise 1.Two expertise 2. 3 or more expertise |  | |  | | 2 |
| **A6a** | **National plan on obesity prevention/promotion of healthy lifestyle** | a. Are there on-going programmes / campaigns as part of a National Plan on obesity prevention / promotion of healthy lifestyle? | |  | a. No/Don't know -> *skip the next question* b. Yes-> *go to the next question* |  | |  | | 0 |
| **A6b** | **Collaboration with existing programmes of National plan** | b. Does your programme fit in that plan? | |  | 0. We do not fit in the plan  2. we are part/fit in the plan |  | |  | | 2 |
| **A7a** | **Communities reached** |  | |  |  | Descriptive | | NOT SCORED | | |
| **A7b** | **People reached** |  | |  |  | Descriptive | | NOT SCORED | | |
| **A7c** | **Target groups** |  | |  |  | Descriptive | | NOT SCORED | | |
|  | **A. TOTAL** | | | | | | |  | | 8 |
| **B. POLITICAL INVOLVEMENT** | | | | | | | | | | |
| **CODE** | **ELEMENT** | **INTERVIEW QUESTION** | | **ANSWER** | **SCORING CATEGORIES** | **REASERCHERS’ COMMENT** | **POINTS SCORED** | | | **MAX POINTS** |
| **B1** | **Formal agreement** | Is there a formal agreement to the programme at National/regional level with political structure? | |  | 0. No 2. Yes |  |  | | | 2 |
| **B1a** | **Type of contribution** | What do(es) political partner(s) provide to the XXX programme ? | |  | 0. No contribution 1.Financial/expertise/in kind contribution/ networking advocacy-visibility/institutional support  2. three or more of 1 (incl. financial/expertise/in kind) |  |  | | | 2 |
| **B1b** | **Person in charge of Political partnerships establishment and management** | Who is responsible for creating/handling the political commitment in your team? What are his/her tasks regarding it? | |  | 0. Nobody 1. someone but not a specific person of the team 2. one or more clearly identified people |  |  | | | 2 |
| **B2a** | **Municipal support** | Is the programme supported within the municipality? | |  | 0. No 1. sometimes 2.yes always |  |  | | | 2 |
| **B2b** | **Type of municipal contribution** | *How does this translate in practice (structures, organisations, human resources, funds)* | |  | 0. No contribution 1.Financial/expertise/in kind contribution/structures/ organisations/ human resources 2. More than two of 1. |  |  | | | 2 |
| **B3** | **Advocacy of political partners NATIONAL LEVEL** | Do political partners actively advocate the programme? How? | |  | 0. No 1.A little (involved but passive) 2. Yes (pro-active) |  |  | | | 2 |
|  | **Add: Advocacy of political partners LOCAL LEVEL** |  | |  | 0. No 1.A little (involved but passive)/some pro-active, some not 2. Yes (pro-active) |  |  | | | 2 |
| **B4** | **Programme reputation within political structures NATIONAL LEVEL** | What do the National/Regional political representatives think about the programme? (supporters, neutral, against) | |  | a. Against b. Neutral c. Positive |  | NOT SCORED | | | |
| **ADDITIONAL** | **Opinion of political representative on programme LOCAL LEVEL** |  | |  | a. Against b. Neutral c. Positive |  | NOT SCORED | | | |
| **B5** | **Political representative** | Are you working directly with a political representative representing the programme? | |  | 0. No 2. Yes |  |  | | | 2 |
| **B5a** |  | Who? | |  |  |  | NOT SCORED | | | |
| **B6** | ***Intersectoral contribution*** | *Do the other sectors of the municipality contribute to the XXX programme?* | |  | 0. Not at all (or barely) 1. contribution in some of the communities 2. contribution in the majority of the communities |  |  | | | 2 |
| **B6a** | ***Intersectoral collaboration*** | *How? Is there cooperation between these sectors (Intersectoral cooperation)?* | |  | 0. Not at all (or barely) 1. Intersectoral in some of the communities 2. Intersectoral in the majority of the communities | Depends on the municipality rather than on the programme | NOT SCORED | | | |
| **B6b** | ***Which sectors?*** | Which ones? | |  | 0. 1 sector 1. 2-3 sectors 2. More than 3 sectors |  |  | | | 2 |
| **B6c** | ***Elected representatives (aldermen/decision makers) contribution*** | Do elected representatives of the municipality contribute to the programme? | |  | 0. No  1. Sometimes 2.Yes always |  |  | | | 2 |
| **B7a** | **Type of communication with Political partners** | How* do you communicate the progress of the programme to your political partners? | |  | 0. No communication 1. Some of the list **excl.** face-to face communication  2. Some of the list **incl.** face-to face communication | *****List face-to-face meeting, telephone, skype, email, letter |  | | | 2 |
| **B7b** | **Frequency of Communication with Political partners** |  | |  | 0. 1 time / year or less  1. 2-3/year 2. more than 3/year |  |  | | | 2 |
| **B9a** | **Satisfaction** | Are you satisfied with the established political commitment? | |  | a. No  b. It could be better c. Yes, satisfied |  | NOT SCORED | | | |
| **B9b** | **Needs assessment** | What is needed to progress? | |  |  |  | NOT SCORED | | | |
| **B. TOTAL** | | | | | | |  | | | 26 |
| **C. PUBLIC AND PRIVATE PARTNERSHIPS** | | | | | | | | | | |
| **CODE** | **ELEMENT** | **INTERVIEW QUESTION** | **ANSWER** | | **SCORING CATEGORIES** | **RESEARCHERS’ COMMENT** | **POINTS SCORED** | | | **MAX POINTS** |
| **C1** | **Involvement of private partnerships** | Are any there PPPs involved? |  | | 0.No  1.Yes, occasionally  2.Yes, constantly |  |  | | | 2 |
| **C1a** | **PPP management in the team** | Who is responsible for creating/handling the PPPs in your team? What are his/her tasks regarding it? |  | | 0. Nobody 1. Someone but not a specific person of the team 2. One or more clearly identified people |  |  | | | 2 |
| **C1b** | **Type of involvement** | How PPP is being applied in the national/regional level? What does it mean for the programme (parties involved in the agreement – NGO and communication agency, national/local authorities and private partners, government and NGO/for-profit organisation? |  | |  |  | NOT SCORED | | | |
| **C1c** | **Financing PPP activities** | How is this activity (e.g. time/materials/personnel spent for recruitment and management) financed? |  | |  |  | NOT SCORED | | | |
| **C1d** | **Facilitators and Barriers** | Helping factors/barriers for the development of PPP? |  | |  |  | NOT SCORED | | | |
| **C2** | **Knowledge on PPPs** | Has knowledge been acquired for the development and management of PPP, and if so how? |  | | 0. No 1. Short training 2. Existing expertize or experience |  |  | | | 2 |
| **C3** | **Government opinion on PPP** | What is the position of the National/Regional government regarding PPP? |  | | a. Negative/ reluctant  b. Neutral  c. Positive |  | NOT SCORED | | | |
| **C5a** | **NATIONAL LEVEL- Private** | Which are your actual partners? |  | | 0. 0 1. 1-3  2. more than 3 |  |  | | | 2 |
| **C5b** | **NATIONAL LEVEL - Public/Non for profit** | Which are your actual partners? |  | | 0. 0 1. 1-3  2. More than 3 |  |  | | | 2 |
| **C5ci** | **Potential Private Partners** |  |  | |  |  | NOT SCORED | | | |
| **C5cii** | **Potential Public Partners** |  |  | |  |  | NOT SCORED | | | |
| **C6** | **PPP CHARTER** |  |  | | 0. No charter 1. Some conditions / partner 2. Charter |  |  | | | 2 |
| **C7a** | **Contribution of private partners** | How do these parties contribute to the programme? |  | | 0. No contribution 1.Financial/expertise/in kind contribution/ networking advocacy-visibility/institutional support / human resources  2. 3 or more of 1^st^ category (incl. financial/expertise/in kind) |  |  | | | 2 |
| **C7b** | **Contribution of public partners** | How do these parties contribute to the programme? |  | | 0. No contribution 1.Financial/expertise/in kind contribution/ networking advocacy-visibility/institutional support / human resources  2. three or more of 1 (incl. financial/expertise/in kind) | Within advocacy visibility is considered |  | | | 2 |
| **C8** | **Motive for partnership** | Why did these partners join the programme? a. How did you convince them? b. What is the advantage for them to join the programme? / Good reputation? / Health of employees? / corporate social responsibility / other)? |  | |  |  | NOT SCORED | | | |
| **C9** | **Communication with Private partners (frequency of contacts)** | How often do you meet your private partners and on what occasions* |  | | 0. 1 time / year or less (no face to face contact) 1. 2-3/year (incl. 1 face to face contact) 2. more than 3/year (incl. 1 face to face contact) | * List: face-to-face meeting, events |  | | | 2 |
| **C10** | **Satisfaction** | Are you satisfied with the established PPPs? |  | | a. No  b. It could be better c. Yes, satisfied | NOT SCORED |  | | |  |
| **C10i.** | **Needs assessment** | What is needed to progress? |  | |  |  | NOT SCORED | | | |
| **C. TOTAL** | | | | | | |  | | | **16** |
| **D. DEVELOPMENT OF INTERVENTIONS AND CAMPAINGS** | | | | | | | | | | |
| **CODE** | **ELEMENT** | **INTERVIEW QUESTION** | **ANSWER** | | **SCORING CATEGORIES** | **RESEARCHERS’ COMMENT** | **POINTS SCORED** | | | **MAX POINTS** |
| **D1** | **Are there interventions/campaigns** | Do you develop and implement interventions and/or campaigns? Specify |  | | 0. No  1. Occasionally 2. Systematically |  | 2 | | | 2 |
| **D1a** | **Level of implementation of intervention** | At what level? |  | | Descriptive |  | NOT SCORED | | | |
| **D2** | **Knowledge for developing interventions/**  **campaigns** | Has knowledge been acquired regarding the design and deployment of interventions and/or campaigns? Specify. a. Existed expertise or training(s)? Could you explain? |  | | 0. No  1. Short training/short-term experience (less than 4 years and developed few interventions) 2. Expertize-appointed experts /long term experience (more than 4 years and developed various interventions) |  |  | | | 2 |
| **D3a** | **Age groups of interventions / campaigns** | Which are the target groups of your interventions / campaigns? specify |  | | 0. One of the list 1. 2 target groups of the list 2. All 3 target groups of the list | List: Children 0-3, children 4-12, adolescents 13-18 |  | | | 2 |
| **D3b** | **Parents as target groups** |  |  | | 0. No  1. Occasionally 2. Constantly (each campaign) | We need to know if there are specific interventions/actions towards parents-Account for it for 2nd appraisal |  | | | 2 |
| **D3c** | **Intermediate target groups** |  |  | | 0. None  1. Occasionally 2. Constantly |  |  | | | 2 |
| **D3d** | **Local stakeholders considered as target groups** | Do you consider the local stakeholders participating in the interventions and campaigns as target groups? |  | | 0. No  1. Occasionally 2. Constantly (each campaign) |  |  | | | 2 |
| **D4** | **Target group analysis** | Has a target group analysis been done? If yes, how did you use it? |  | | 0. No  1. Occasionally/partly 2. Constantly (each campaign) | Here analysis of the final target group assessed; social marketing in the field |  | | | 2 |
| **D5a** | **Multi-disciplinary team for the planning phase** | Could you please describe the process of the **planning** of an intervention? a. Who is responsible for this? |  | | 0. One expertise 1.Two expertise 2. 3 or more expertise |  |  | | | 2 |
| **Additional** | **commitment of the team in planning phase** | Do they work full time or part time? |  | | 1. Part time /Some part time, some full time/ on demand  2. Everyone full time |  |  | | | 2 |
| **D5b** | **Following a process to select a theme** | *How is the theme decided?* On what basis (needs analysis, focus groups, ITVs, feedback from local project managers). Please give details of the process. |  | | 0. No specific basis  1. Based on scientific knowledge or assessments on the local level 2. Based on scientific knowledge and assessments on the local level |  |  | | | 2 |
| **D5c** | **Multi-stakeholder involvement in the planning phase** | Who else is involved and what are the specific tasks? |  | | 0. No  1. Yes occasionally 2. Yes constantly |  |  | | | 2 |
| **D5ci** | **Final target group involvement in the planning phase** | Who else is involved and what are the specific tasks? |  | | 0. No  1. Yes occasionally 2. Yes constantly |  |  | | | 2 |
| **D5cii** | **Can Private partners intervene in the contents?** | Any private partners? |  | | 0. Yes 1. No |  |  | | | 1 |
| **D5d** | **Satisfaction regarding the planning** | Are you satisfied with the overall process for planning the interventions and campaigns? What is needed to progress? |  | | a. No  b. It could be better c. Yes, satisfied |  | NOT SCORED | | | |
| **D5di** | **Need assessment** | What is needed to progress? |  | |  |  | NOT SCORED | | | |
| **D6b** | **Existence of a social marketing team OR Expert** | Who is developing the tools? Form contents to graphic design and printing |  | | 0. No  1. Yes, one person 2. Yes, a team |  |  | | | 2 |
| **D6c** | **Validation of the campaigns contents** | Is there anyone validating the contents of the tools/intervention? Please give details |  | | 0. No  1. Yes, by one expert (e.g. dietician) 2. Yes, by the SAB or more experts |  |  | | | 2 |
| **D6e** | **Training of the Local Manager** | Is there any training of the people involved in the field (form LPM to volunteers)? |  | | 0. No  1. For some activities/campaigns 2. Yes, systematically |  |  | | | 2 |
| **D5f** | **Satisfaction regarding the implementation** | Are you satisfied with the overall process for **implementing** the interventions and campaigns? |  | | a. No  b. It could be better c. Yes, satisfied |  | NOT SCORED | | | |
| **D5fi** | **Programme Need assessment** | What is needed to progress? |  | |  |  | NOT SCORED | | | |
| **D7** | **Tools developed/used** | What tools do you develop for your target groups? (e.g. intervention guide, poster, leaflet, book, recipe sheet) - |  | | 0. None  1. Methodological or communication tools 2. methodological and communication tools | methodological tools (e.g. action sheet, guide) Communication tools (e.g leaflet, poster, whatever increase knowledge) |  | | | 2 |
| **D8** | **Use of tools from other programmes** | If there are on-going programmes / campaigns on childhood obesity prevention or on other related public health issues, do you make use of their tools? How? |  | |  | Not a Social Marketing element | NOT SCORED | | | |
| **D9** | **Use of experience of local actors** | Do you use the experience of the local coordination teams/ actors directly involved for the design of future interventions/campaigns? A. If yes, how (the actions they have implemented, their methodology, the tools they have developed, their feedback on the tools etc)? |  | | 0. Never 1. Sometimes 2.Constantly |  |  | | | 2 |
| **D10a** | **Diversity of the themes** | What do your interventions/ campaigns (specify) include in terms of:  a. Themes: Food habits/physical activity/ other |  | | 0. 1 theme (e.g. only PA) 1. 2 themes (e.g. PA and food habits) 2. More than 2 themes |  |  | | | 2 |
| **D10ai** | **Changing of the environment** |  |  | | 0. None 1. one macro/micro-environmental change 2. both macro/micro-environmental change or more on than one in any level | micro- (e.g. family, culture) and macro-level considered equally important; though more stakeholders involved in macro level |  | | | 2 |
| **D10b** | **Diversity of the activities** | What do your interventions/ campaigns (specify) include in terms of: b. Activities: organization of events, workshops, PR/ other |  | | 0. single method/approach for short term 1. single method/approach for long term 2. Synergy of methods with interventions |  |  | | | 2 |
| **ADDITIONAL** | **Number of activities implemented / month** |  |  | | 0. Less than 3 1. 2 to 5  2. More than 5 |  |  | | | 2 |
| **D. TOTAL** | | | | | | |  | | | 43 |
| **E. COMMUNICATION** | | | | | | | | | | |
| **CODE** | **ELEMENT** | **INTERVIEW QUESTION** | **ANSWER** | | **SCORING CATEGORIES** | **RESEARCHERS’ COMMENT** | **POINTS SCORED** | | | **MAX POINTS** |
| **E1** | **Training of the coordination Team** | Has knowledge been acquired on communication / PR activities? |  | | 0. No 1. Short training 2. Expertize/assigned to expert |  |  | | | 2 |
| **E2** | **Communication materials and channels** | What communication materials and channels* do you use? |  | | 0. none 1. 1 to 4 channels 2. More than 4 | *Website, Newsletter, Press releases, Press events, One-to-one direct communication (journalists, political, scientific, private partners), Newspapers, TV, social media, Web TV, Radio, Facebook, Twitter, Google +, Google groups, other |  | | | 2 |
| **E3** | **Communication responsibility** | Who is responsible. for the communication on the programme |  | |  | For information | NOT SCORED | | | |
| **E4** | **Involvement of PPP in communication activities** |  |  | |  | There can be advantages and inconvenience | NOT SCORED | | | |
| **E5** | **Existence of a communication plan** | Is there a communication plan? If yes, how often is it being set-up? |  | | 0. No 1. We answer when there is an opportunity  2. Communication plan |  |  | | | 2 |
| **E6** | **Evaluation of the PR campaigns** | Do you evaluate your PR communication? |  | | 0. No 1. Yes light (press clips) 2. Yes consistent (number of people reached, ROI) |  |  | | | 2 |
| **E6a** | **PR Results** |  |  | |  |  | NOT SCORED | | | |
| **E6b** | **Satisfaction with communication results** | b. Are you satisfied with these results? Why? |  | | a. No  b. It could be better c. Yes, satisfied |  | NOT SCORED | | | |
| **E. TOTAL** | | | | | | |  | | | 8 |
| **F. SCIENTIFIC APSECTS** | | | | | | | | | | |
| **CODE** | **ELEMENT** | **INTERVIEW QUESTION** | **ANSWER** | | **SCORING CATEGORIES** | **COMMENT** | **POINTS SCORED** | | | **MAX POINTS** |
| **F1a** | **Existence of scientific support** | Did you create a scientific advisory board or individual experts are collaborating occasionally, or both? Please explain. |  | | 0. None 1. Expert consultation on demand 2. SAB |  |  | | | 2 |
| **F1b** | **Fields of expertize** | Who is part of it? What are their areas of expertise? |  | | 0. One expertise 1.Two expertise 2. 3 or more expertise |  |  | | | 2 |
| **F1c** | **Role of SAB / experts** | What are their responsibilities* within the programme? |  | | 0. Less than 2 (from list) 1. 2 to 3 (from list) 2. More than 3 (from list) | *List:  Tools’ contents, Tools’ validation, Evaluation, publication, data collection, data analyses, programme design and implementation, applications for funds |  | | | 2 |
| **F1d** | **Scientific Spokesperson** | Do you have scientific spokespersons? Who? |  | | 0. No 1. Yes, but not a specific person 2. Yes, one or more clearly identified spokesperson |  |  | | | 2 |
| **F1e** | **Meeting frequency with scientific experts** | How often do you meet the scientific experts |  | | 0. 1 per year or less 1. 2 to 3 per year 2. More than 3 per year |  |  | | | 2 |
| **F1f** | **How is scientific support financed** |  |  | |  |  | NOT SCORED | | | |
| **F1g** | **Satisfaction** | How do you feel about this collaboration? |  | | a. No  b. It could be better c. Yes, satisfied |  | NOT SCORED | | | |
| **F2** | **Training of the coordination team on sc. Aspects** | Have knowledge been acquired on the scientific aspects of the programme (understanding obesity, intervention protocol, evaluation) and if so how? |  | |  |  | NOT SCORED | | | |
| **F3** | **Systematic evaluation approach** | Does an evaluation take place? |  | | 0. No 1. Yes, but not in representative sample/insufficient/sometimes 2. Yes, in a representative sample/ sufficient/always |  |  | | 2 | |
| **F3a** | **Evaluation framework** | Is there an evaluation framework? |  | | 0. No 2. Yes |  |  | | 2 | |
| **F3ai** | **Evaluation methodology** | Does it follow a specific methodology (e.g a logic model, definition of SMART objectives)? |  | | 0. No  2. Yes |  |  | | 2 | |
| **F3b** | **Who responsible for the evaluation** | d. Who is responsible for the evaluation? |  | | 0. Nobody 1. No scientific discipline responsible 2. Scientific discipline (s) responsible |  |  | | 2 | |
| **F4ai** | **Is the central coordination level evaluated** | Are processes evaluated? |  | | 0. No 1. Yes, Sometimes/partly 2. Yes, Systematically |  |  | | 2 | |
| **F4aii** | **Is the local coordination level evaluated** | Are processes evaluated? |  | | 0. No 1. Yes, Sometimes/partly 2. Yes, Systematically |  |  | | 2 | |
| **F4aiii** | **Is the setting (actions) evaluated** | Are processes evaluated? |  | | 0. No 1. Yes, Sometimes/partly 2. Yes, Systematically |  |  | | 2 | |
| **F4bi** | **Are the children/families behaviour change evaluated** | Are effects evaluated? |  | | 0. No 1. Only once 2. Yes |  |  | | 2 | |
| **F4bii** | **BMI evaluation** | Are effects evaluated? |  | | 0. No  1. Only once 2. Yes |  |  | | 2 | |
| **F5** | **Frequency of evaluation** | At what points does evaluation take place? |  | | 0. No evaluation 1. After/one measurement 2. Before and after/ More regularly |  |  | | 2 | |
|  | **ADD Against a control group?** |  |  | | 0. No control group 2. Yes |  |  | | 2 | |
| **F6** | **Satisfaction** | Are you satisfied with the evaluation process of your programme (what is evaluated, funding, feasibility)? |  | | a. No  b. It could be better c. Yes, satisfied |  | NOT SCORED | | | |
| **F6i** | **Need assessment** | What is needed to progress? |  | |  |  | NOT SCORED | | | |
| **F7** | **Results** | Do you have implementation and evaluation results? |  | | 0. No 1. In process 2. Yes | Judging for the overall programme-existence of results |  | | 2 | |
| **F7a** | **Dissemination in communities and stakeholders** | How do you use them (scientific publication, dissemination, other use) |  | | 0.Not at all  1.Yes to the communities or stakeholders  3. Yes to communities and stakeholders |  |  | | 2 | |
| **F7b** | **scientific publications (peer review)** | b. Do you have scientific publications? |  | | 0.No 1. Under review  2.Yes |  |  | | 2 | |
| **F8** | **Participation to scientific events / year** | What scientific events do you participate in and how (poster, presentation, workshop…)? |  | | 0. None 1. Yes 1 to 3 2. Yes more than 3 | score "yes or no" when number of conferences is missing |  | | 2 | |
| **F9** | **Attendance to sc. Events** |  |  | |  |  | NOT SCORED | | | |
| **F10** | **% Budget for Evaluation** | % Budget for Evaluation |  | | 0. Less than 5% 1. 5 to 10% 2. More than 10% |  |  | | 2 | |
| **F. Total** | | | | | | |  | | **38** | |
| **TOTAL** | | | | | | | | | | |
